# Utilisation and financial protection for hospital care under publicly funded health insurance in three states in Southern India

**DOI:** 10.1186/s12913-019-4849-8

**Published:** 2019-12-27

**Authors:** Samir Garg, Sayantan Chowdhury, T. Sundararaman

**Affiliations:** 1 State Health Resource Centre, Chhattisgarh, Raipur, India; 2 Formerly Professor, School of Health Systems Studies, Tata Institute of Social Sciences, Mumbai, India

**Keywords:** Insurance, Coverage, Financing, India, Healthcare, Utilisation, Catastrophic expenditure, Out-of-pocket, Purchasing

## Abstract

**Background:**

Many LMICs have implemented Publicly Funded Health Insurance (PFHI) programmes to improve access and financial protection. The national PFHI scheme implemented in India for a decade has been recently modified and expanded to cover free hospital care for 500 million persons. Since increase in annual cover amount is one of the main design modifications in the new programme, the relevant policy question is whether such design change can improve financial protection for hospital care. An evaluation of state-specific PFHI programmes with vertical cover larger than RSBY can help answer this question.

Three states in Southern India - Andhra Pradesh, Karnataka and Tamil Nadu have been pioneers in implementing PFHI with a large insurance cover.

**Methods:**

The current study was meant to evaluate the PFHI in above three states in improving utilisation of hospital services and financial protection against expenses of hospitalization. Two cross-sections from National Sample Survey’s health rounds, the 60th round done in 2004 and the 71st round done in 2014 were analysed. Instrumental Variable method was applied to address endogeneity or the selection problem in insurance.

**Results:**

Enrollment under PFHI was not associated with increase in utilisation of hospital care in the three states. Private hospitals dominated the empanelment of facilities under PFHI as well as utilisation. Out of Pocket Expenditure and incidence of Catastrophic Health Expenditure did not decrease with enrollment under PFHI in the three states. The size of Out of Pocket Expenditure was significantly greater for utilisation in private sector, irrespective of insurance enrollment.

**Conclusion:**

PFHI in the three states used substantially larger vertical cover than national scheme in 2014. The three states are known for their good governance. Yet, the PFHI programmes in all three states failed in fulfilling their fundamental purpose. Increasing vertical cover of PFHI and using either ‘Trusts’ or Insurance-companies as purchasers may not give desired results in absence of adequate regulation. The study raises doubts regarding effectiveness of contracting under PFHIs to influence provider-behavior in the Indian context. Further research is required to find solutions for addressing gaps that contribute to poor financial outcomes for patients under PFHI.

## Introduction

Nations of the world have committed to the target of Universal Health Coverage (UHC) under Sustainable Health Goals [1]. UHC is aimed at improving access and financial protection for healthcare [2]. Many LMICs have initiated Publicly Funded Health Insurance (PFHI) programmes in order to achieve the above objectives [3–7]. PFHI has been implemented in India for more than a decade now [8–10]. It forms the focus of current healthcare policies in India. The central government has launched a large PFHI programme called Pradhan Mantri Jan Arogaya Yojana (PMJAY) that promises free hospital care for 500 million persons. The merits or disadvantages of the PFHI-based policy are being vigorously debated in India and also internationally [11–17].

PFHI programmes in India are focused on covering hospital care. Most of the evidence on impact of PFHI in India indicates that it has failed to achieve financial protection [8, 18–29]. A smaller set of studies have reported reduction in Out of Pocket Expenditure (OOPE) due to PFHI [9, 30, 31]. Some studies have reported increase in utilisation of hospital-care due to PFHI [9, 18, 19, 22]. Other studies did not find increase in utilisation of hospital-care due to PFHI [21, 24].

PMJAY aims to build on the base provided by Rashtriya Swasthya Bima Yojana (RSBY), the national PFHI scheme implemented by many states during 2008 to 2018. While RSBY had a vertical cover of INR 30,000 (around 400 USD) annual sum assured, PMJAY has a much bigger cover of INR 500,000 (around 7000 USD) per family [11, 32, 33]. Some of the evaluations of RSBY have suggested that the limited sum covered could be a factor in its inability to protect from catastrophic expenditure [18, 19, 21]. Since a 17 fold increase in vertical cover is the main change in PFHI design brought in by PMJAY, the relevant policy question is whether larger vertical cover can improve financial protection for hospital care under PFHI. An evaluation of state-specific PFHI programmes that have implemented a vertical cover larger than RSBY can help answer this question. Three states in Southern India - Andhra Pradesh, Karnataka and Tamil Nadu which were pioneers in India in initiating PFHI programmes of their own. They differed from RSBY in terms of having a vertical cover around five times bigger than what RSBY offered [10]. Further, there are differences in benefit-packages and implementation arrangements across PFHI schemes in different states in India. This suggests a need to examine performance of PFHI state by state [10, 21, 22].

PFHI, also known as Government Sponsored Health Insurance, is the term we use to denote the new wave of National or State schemes that started in India after 2006 [10]. The schemes are usually meant to provide specific risk coverage for the people outside formal employment. Each state decides a benefit package with a list of services and pre-defined prices. Services are provided by empanelling public as well privately owned hospitals. The schemes are implemented in either of the two ways:

- Insurance Model: State contracts an insurance company and pays it an annual premium per household enrolled. The premium is paid on behalf of the eligible households. The state allocates budget to bear the cost of premium. The Insurance Company empanels hospitals and reimburses them claim amount for cases served according to an agreed upon rate. Tamil Nadu is such a model. It used an insurance company as purchaser
- Trust Model: State sets up a ‘Trust’, which is a government owned autonomous institution to handle the above tasks. Hospitals are reimbursed against claims made by the Trust, for which state allocates a budget to ‘Trust’. Andhra Pradesh and Karnataka used a ‘Trust’ as purchaser organization.

In terms of statistical methods, an important problem in evaluating insurance is of self-selection or endogeneity when enrollment in insurance is non-random. Endogeneity is caused by issues including unobserved variables and simultaneity [6, 7, 34]. Not many evaluations of PFHI in India have taken endogeneity into account. Failure to address endogeneity can distort results of regression. Recent literature on PFHI in India acknowledges this gap and recommends new evaluations that address endogeneity [9, 21, 35].

The current study was aimed at answering the following two questions for each of the three states (Andhra Pradesh, Karnataka and Tamil Nadu), using methods that can address the problem of endogeneity:

- Did enrollment under PFHI lead to increase in utilisation of hospital-care?
- Did enrollment under PFHI lead to reduction in OOPE and incidence Catastrophic Health Expenditure (CHE) for hospital-care?

### Background description of PFHIs in Andhra Pradesh, Karnataka and Tamil Nadu

#### PFHI in Andhra Pradesh

The PFHI scheme in Andhra Pradesh was named ‘Rajiv Arogayasri’ (RAS) at the time of its launch in 2007 and later changed to ‘NTR Vaidya Seva’. The objectives of the PFHI as articulated by state in 2013 were – “To improve equity of access to ‘Below Poverty Line’ (BPL) families to quality tertiary medical care both by strengthening the Public Hospital infrastructure as well as through purchase of quality private medical services to provide financial support for catastrophic health needs.” [36].

According to the BPL estimation by central government, the proportion of BPL in 2015 was 29.6, 33.3 and 29.4% in Andhra Pradesh, Karnataka and Tamil Nadu respectively, whereas the national average was 37.2% [37]. However, in Andhra Pradesh around 80% of the families were classified as BPL by the state government and it considered all of them as enrolled in PFHI [38]. The per capita income (at 2013–14 prices) in Andhra Pradesh in 2013–14 was INR 82,870, which was lower than Karnataka (INR 118,829) and Tamil Nadu (INR 116,236) [39]. There was no restriction on the number of members per family who could enroll. PFHI programme had an annual sum assured of INR 200,000 in year 2014. Its benefit package stipulated free care or fully cashless benefit including all direct medical expenses and transportation. It covered 942 procedures and 125 follow-up packages, mainly for surgical or tertiary hospital-care. There were 586 hospitals empanelled under the scheme in 2014, of which 460 (78%) were private hospitals [40].

The state created a Trust called Rajiv Arogayasri Trust to act as purchaser. Initially an insurance company was also used as intermediary for part of PFHI operations in some districts. From 2013 onwards, the entire purchasing role across the state was handled by the Trust. The leadership of the Trust consisted of government officials from the state’s health department, and its day to day functioning was managed by professional staff. Though an entirely government owned body, the creation of a trust provided it with considerable autonomy that was essential to manage a wide range of operational decisions. The empanelment of private or public hospitals was decided by the Trust. The hospitals sent claims for payment to the Trust through electronic means. Hospitals were paid per episode basis, at prices stipulated in the benefit package. The Trust hired the services of Third Party Administrators (TPAs) to help it in managing the operations of the scheme. The scheme also deployed staff called ‘Arogaya Mitras’, who acted as first contact for the patients seeking care. The scheme stipulated ‘pre-authorisation’ for all services provided under its cover [9, 38]. There is considerable literature on the scheme but most of it relates to its early phase, between 2007 and 2012 [9, 29–31, 41, 42].

#### PFHI in Karnataka

Karnataka started “Vajpayee Arogyasri scheme” (VAS) in 2009 to provide health insurance coverage for BPL families. Initially the VAS scheme was implemented in five pilot districts and by 2012 it was rolled out across the entire state [43, 44]. All BPL families were automatically considered to be enrolled in the scheme. A maximum of five members per family were eligible to seek treatment in a year.

The maximum insurance coverage amount was INR 200,000 (INR 150,000 per family per year with additional buffer of INR50000 for specific needs). Its benefit package stipulated free care or fully cashless benefit and was focused on hospital care of tertiary nature [43]. The provider payments were based on the predefined package rates for 459 listed procedures and 50 follow-up packages.

A Trust set up by the state managed the scheme with help of a TPA [43]. The Trust was an autonomous body constituted by the government with deputed government officers in leading positions and professional staff managing the work. The Trust was responsible for managing hospital empanelment, pre-authorizations, claim processing, and payments to hospitals [44]. In 2014, services were provided by 144 hospitals, around 85% of them were private [45]. The existing literature on VAS relates to its early stages, till 2012 [31, 43, 46].

#### PFHI in Tamil Nadu

Tamil Nadu started its PFHI scheme in year 2009 and this was known as Chief Minister Kalaignar’s Health Insurance Scheme (CMKHIS) [47]. In 2012, with a change in government, the PFHI was renamed as the Tamil Nadu Chief Minister’s Comprehensive Health Insurance Scheme (CMCHIS) [47]. Its stated aim was to provide financial risk protection to poor and other vulnerable population of the state against expensive therapeutic and surgical health conditions [48]. The main objective of the CMCHIS was to provide cashless medical and surgical treatment in all government and empanelled private hospitals [48]. The scheme targeted poor families. The eligibility criterion for enrollment was annual household income of INR 72,000 or less [49]. The maximum insurance coverage amount was INR 400,000 per family over 4 years. Its benefit package stipulates free care or fully cashless benefit and is focused on hospital care of tertiary nature, covering 1016 procedures [49].

Tamil Nadu Health Systems Society (TNHSS), a body set up by the state, was responsible for the monitoring and implementation of the scheme. TNHSS selected an Insurance Company, which with help of TPAs carried out enrolment of eligible households, empanelment of hospitals and payments to empanelled hospitals. Liaison Officers were deputed at each empanelled hospital for helping patients in enrollment and availing benefits of the scheme [49]. Out of total 771 hospitals empanelled, 80% were private hospitals [49]. There is no quantitative impact evaluation study available on PFHI in Tamil Nadu and the existing literature is on implementation processes [47, 49].

## Methods

Some of the recent systematic reviews of PFHI in LMICs have provided detailed methodological recommendations for examining effects of health insurance [6, 7]. A study evaluating PFHI in China has shed light on useful statistical methods [34]. The Instrumental Variable (IV) method has been recommended as a robust solution to potential problem of endogeneity [6, 7, 34, 50, 51]. IV method has been considered more suitable than Difference in Difference (DID) and Propensity Score Matching for addressing endogeneity bias due to unobserved variables [7, 50]. IV method has been applied in evaluations of impact of PFHI programmes on OOPE and CHE in Mexico and Ghana [52, 53]. IV method has been found effective in addressing endogeneity in cross-sectional datasets [7, 34]. There is a recommendation that using observations of two different times is ideal, with one measurement before the insurance scheme began [6].

The National Sample Survey (NSS) of India offers a suitable opportunity in form of its two cross-sectional datasets. The 60th round of NSS provides data on hospital care and OOPE for year 2004, which was before PFHI programmes were introduced in any state of India [54]. The 71st round of NSS in 2014 provides data after PFHI schemes were in operation for several years [55, 56]. By 2014, each of the three states being studied - Andhra Pradesh, Karnataka and Tamil Nadu, had implemented PFHI for five or more years and with a vertical cover (annual sum assured) exceeding INR 100,000 per family.

The NSS follows two-stage stratified sampling. A detailed note on the sample design is available in NSS documents and datasets [54, 56–58]. NSS survey in 2014 covered 10,636 individuals in Andhra Pradesh, 14,727 in Karnataka and 16,090 in Tamil Nadu, as a weighted sample of their rural and urban populations [55, 56]. The corresponding figures in 2004 survey were 22,387, 16,986 and 21,294 individuals respectively [57]. Since one of the main objectives of the study was to detect the change in financial protection for hospital care, we also needed adequate number of hospitalization episodes in the sample. For a detectable difference of 5% at 95% confidence and a design effect of 1.5, we calculated a requirement of around 576 hospitalization episodes in each of the three states. The actual number of hospitalization episodes covered for Andhra Pradesh, Karnataka and Tamil Nadu in the survey sample was 2305, 1455 and 2422 respectively for year 2004 and it was 2288, 2729 and 3646 for year 2014 [54, 56–58]. The size of sample available was therefore adequate to detect difference of 5%.

OOPE amounts for 2004 were adjusted at 2014 prices for valid comparison, as done by a recent study using the same datasets [59]. For the above adjustment, price deflators for rural (agricultural labour) and urban areas (industrial workers) were used [60].

Financial Protection was measured in terms of Catastrophic Health Expenditure (CHE) as proposed by Wagstaff and Doorslaer [61]. Out of Pocket Expenditure (OOPE) was calculated for each episode by adding medical expenses and expenses on transportation and deducting any cash-reimbursements received by the patient. The survey collected data on usual monthly consumption expenditure and it was multiplied by twelve to calculate the Usual Annual Consumption Expenditure. Recent studies analyzing the (NSS) dataset have also used the same procedure for calculating Annual Household Consumption Expenditure [20, 22, 24]. Thresholds of 10, 25 and 40% of concerned household’s Annual Consumption Expenditure were taken for CHE and named CHE10, CHE25 and CHE40 respectively. The list of variables in the study is given in Additional file 1.

The survey data was analysed using STATA V.14. Instrumental Variable (IV) method was used in the multivariate analysis. We applied Two-step least square (2sls) for OOPE and Two-step IV Probit model for Utilisation and CHE [62]. Some studies on impact of insurance using IV method have applied 2sls for OOPE and Control Function tests like two-stage residual inclusion for CHE [52, 53]. For robustness, we repeated the IV regressions for CHE using 2sls and also Two Step Control Function test available in STATA for endogenous covariates. Other studies have also used comparisons with IV Probit for robustness [51].

A suitable ‘Instrumental Variable’ should satisfy the ‘relevance’ criterion i.e. it should correlate with the explanatory variable, i.e. PFHI-enrollment in this case [7, 63]. Instrumental Variables were selected from amongst - Social Group (Caste), Sex, Place of Residence (Rural/Urban) because earlier studies have reported their association with PFHI enrollment in India [20, 22, 24, 64]. We expect the above variables to be associated with enrollment in PFHI but unlikely to affect OOPE directly. A suitable Instrumental variable should not have a direct impact on the outcome variable [59]. This restriction, also called ‘over-identifying restriction’, is tested by including each subset of instruments in the last stage regressions to see whether these instruments can be justifiably excluded from these regressions. According to literature, that test allows us to evaluate the validity of the model [6, 7, 34, 64]. Wu-Hausman test for 2sls (using command “estat endog”) and Wald test of exogeneity for IV Probit were conducted to test for endogeneity. Over-identification restriction tests (command “weakiv” for two step IV Probit and “estat overid” for 2sls) were applied to check the suitability of Instrumental Variable model chosen [62]. The results of the above tests have been reported along with the regression results. Significance was taken at 95% (*p* < 0.05).

## Results

The sample profile is given in Additional file 2. In 2014, the proportion of respondents who reported being enrolled in any publicly funded health insurance scheme was lower in Karnataka and Tamil Nadu as compared to Andhra Pradesh (Table 1).

**Table 1:** Proportion of individuals enrolled under PFHI (in 2014)

State	Proportion of individuals enrolled in PFHI (%)	95% Confidence Intervals	
Andhra Pradesh	62.6	61.6	63.5
Karnataka	5.2	4.8	5.6
Tamil Nadu	17.8	17.2	18.4

Source: Authors’ Analysis of NSS 2014

### Utilization of hospital care

In Andhra Pradesh, hospitalization rate amongst the PFHI enrolled was slightly lower than the non-insured individuals in 2014. In other states the PFHI-enrolled had slightly higher hospitalization rate than the non-insured. Over the decade hospitalization rates had gone up in all three states (Table 2).

**Table 2:** Annual Hospitalization Episodes per unit population

State	Proportion (%) of individuals who utilized hospital care with 95% Confidence Intervals in ()			
	In 2004	In 2014		
	All	All	Non-insured individuals	PFHI-enrolled individuals
Andhra Pradesh	2.29 (2.09–2.49)	5.58 (5.14–6.01)	5.86 (5.18–6.53)	5.41 (4.84–5.99)
Karnataka	2.23 (2.01–2.46)	4.93 (4.58–5.28)	4.88 (4.53–5.24)	5.76 (4.08–7.43)
Tamil Nadu	3.58 (3.33–3.83)	5.68 (5.32–6.04)	5.55 (5.16–5.94)	6.27 (5.38–7.17)

Source: Authors’ Analysis of NSS 2004 and 2014

The naïve Probit model showed a significant association between hospitalizations and PFHI enrollment in Karnataka but not in other two states (Table 3).

**Table 3:** Coefficients and significance of PFHI-Enrollment variable in Naïve Probit Model for Utilisation (Hospitalisation)

Andhra Pradesh	Karnataka	Tamil Nadu
−0.025	0.191***	−0.022

Significance: ***p < 0.01

IV Probit regression was carried out to find out the determinants of Utilisation i.e. hospitalization (Additional file 3). Enrollment under PFHI was not associated with increase in utilisation in any of the three states. In all three states, the likelihood of hospitalization was greater for older individuals and women. In Karnataka and Tamil Nadu, the likelihood of hospitalization was greater for the educated compared to illiterate and for persons from the poorest households.

#### Choice of provider

Among respondents who experienced hospitalization over the year preceding survey, a majority had utilized private sector. The share of private sector in the PFHI-enrolled hospitalizations was similar to non-insured hospitalizations in the three states (Table 4).

**Table 4:** Proportion of Hospitalisation Episodes in Private Hospitals

State	Insurance status	Total No. of Hospitalisations (2004)	Proportion of episodes in private hospitals (%) with 95% CI in () (2004)	Total No. of Hospitalisations (2014)	Proportion of episodes in private hospitals (%) with 95% CI in ()(2014)
Andhra Pradesh	PFHI Enrolled	–	–	1321	71 (68–73)
	Not Enrolled	2305	70 (68–72)	967	80 (77–82)
Karnataka	PFHI Enrolled	–	–	201	70 (63–76)
	Not Enrolled	1455	65 (62–67)	2528	68 (66–70)
Tamil Nadu	PFHI Enrolled	–	–	681	67 (63–70)
	Not Enrolled	2422	61 (59–63)	2965	61 (59–62)

Source: Authors’ Analysis of NSS 2004 and 2014

### OOPE and financial protection

In all three states, the mean OOPE for utilizing hospital-care in private sector was many times larger than in public sector. This was true for the PFHI-enrolled and also for the non-insured. The mean OOPE was similar for the PFHI-enrolled and for the non-insured hospitalizations (Table 5). From 2004 to 2014, after adjusting for inflation the mean OOPE per hospitalization declined marginally in the private sector and significantly declined in the public sector.

**Table 5:** Mean OOPE for Hospitalisation Episodes (in INR) with 95% CI in ()

State	2004 (At 2014 prices)		2014			
			PFHI Enrolled		Non-enrolled	
	Public Hospital	Private Hospital	Public Hospital	Private Hospital	Public Hospital	Private Hospital
Andhra Pradesh	5042 (4110–5976)	19,657 (17302–22,013)	2864 (1725–4004)	15,827 (14570–17,084)	2355 (1714–2998)	17,934 (15676–20,194)
Karnataka	4511 (3794–5229)	18,085 (16111–20,058)	2888 (1551–4226)	16,121 (12482–19,760)	3556 (3030–4082)	17,873 (16489–19,258)
Tamil Nadu	3291 (1873–4710)	24,637 (20752–28,522)	802 (611–993)	23,966 (21060–26,872)	954 (788–1120)	26,425 (24140–28,711)

Source: Authors’ Analysis of NSS 2004 and 2014 (at 2014 prices)

Calculating for median OOPE, we have similar findings as for mean - the private sector OOPE is many times greater than in public sector and the median OOPE for the PFHI-enrolled was similar to that for non-insured utilization (Table 6). Between 2004 and 2014 median OOPE in the public sector has declined, but in the private sector has gone up, irrespective of PFHI coverage.

**Table 6:** Median OOPE for Hospitalisation Episode (in INR) with 95% CI in ()

State	2004 (At 2014 prices)		2014			
			PFHI Enrolled		Non-enrolled	
	Public Hospital	Private Hospital	Public Hospital	Private Hospital	Public Hospital	Private Hospital
Andhra Pradesh	1660 (1461–1853)	9900 (9020–10,719)	600 (500–850)	10,493 (9894–11,303)	925 (600–1140)	12,130 (10990–13,500)
Karnataka	2027 (1667–2437)	8800 (7700–9612)	1140 (817–1914)	8800 (7239–10,835)	1975 (1700–2250)	10,625 (10000–11,400)
Tamil Nadu	535 (466–629)	10,718 (9602–11,271)	370 (300–500)	15,450 (13900–17,584)	350 (300–400)	15,095 (14000–15,771)

Source: Authors’ Analysis of NSS 2004 and 2014 (at 2014 prices)

#### CHE25 incidence

CHE25 incidence is many times greater for utilisation in private sector as compared to public sector. It is similar for the PFHI-enrolled and for the non-insured (Table 7).

**Table 7:** Proportion of individuals incurred CHE25 for Hospitalisation Episode (%) with 95% CI in ()

State	For PFHI Enrolled in 2014		For Non-enrolled in 2014		2004	
	Public	Private	Public	Private	Public	Private
Andhra Pradesh	2.7 (1.1–4.4)	17.7 (15.3–20.1)	1.7 (0–3.5)	17.1 (14.5–19.8)	6.4 (4.6–8.2)	24.7 (22.6–26.8)
Karnataka	2.2 (0–5.8)	20.0 (13.1–26.9)	3.1 (1.9–4.4)	22.6 (20.6–24.5)	5.1 (3.2–7.0)	23.9 (21.2–26.6)
Tamil Nadu	0 (0–0)	27.2 (23.1–31.4)	0.3 (0–0.6)	29.3 (27.2–31.5)	2.4 (1.5–3.4)	27.4 (25.2–29.7)

Source: Authors’ Analysis of NSS 2014

#### Determinants of size of OOPE and CHE

Initially, naïve models were applied for OOPE and CHE (Table 8).

**Table 8:** Coefficients and significance of PFHI-Enrollment variable in Naïve Models (Ordinary Least Squares (OLS) for OOPE and Probit for CHE10, CHE25 and CHE40)

	Andhra Pradesh	Karnataka	Tamil Nadu
OOPE	− 5374***	− 4064**	2665
CHE10	−0.235***	−0.153	−0.085
CHE25	−0.210***	−0.083	−0.031
CHE40	−0.255***	−0.118	0.090

Significance: *** p < 0.01, **p < 0.05

OLS showed negative association between OOPE and PFHI-enrollment in Andhra Pradesh and Karnataka. The naïve Probit model showed association between PFHI-enrollment and CHE in Andhra Pradesh but none in the other two states.

#### IV Model for OOPE

Amongst the hospitalizations, 2sls regression was carried out to find the determinants of size of OOPE (Additional file 4). Enrollment under PFHI was not associated significantly with the size of OOPE in any of the three states. In all three states, greater OOPE was significantly likely for utilisation in private sector. NCDs or Injuries compared to Communicable diseases and hospitalisations longer than 3 days were also associated with greater OOPE. In Andhra Pradesh and Tamil Nadu, the richest were likely to incur greater OOPE than the poorest quintile. In Karnataka and Tamil Nadu, the better educated were likely to incur greater OOPE than the illiterate.

The above model was repeated for log of OOPE and the results remained similar.

#### IV Model for CHE25

Amongst the hospitalizations, IV Probit regression was carried out for finding the determinants of CHE25 incidence (Additional file 5). It showed that Enrollment under PFHI was not associated significantly with CHE25 incidence in any of the three states. In all the three states, among those who utilized hospital-care, CHE25 was significantly more likely to occur for utilisation in private sector. Diseases other than Communicable diseases and hospitalizations longer than 3 days were also associated with greater chances of CHE25 incidence. The poorest quintile had greater chances of incurring CHE25 compared to other quintiles.

For comparison, IV regression for CHE25 was also carried out through 2sls and through Two Step Control Function treatment but the pattern of results did not change.

#### CHE40 incidence

CHE40 incidence was similar for the PFHI-enrolled and the non-insured (Table 9).

**Table 9:** Proportion of individuals incurred CHE40 for Hospitalisation Episode (%) with 95% CI in ()

State	For PFHI Enrolled in 2014		For Non-enrolled in 2014		For 2004	
	Public	Private	Public	Private	Public	Private
Andhra Pradesh	0.2 (0–0.7)	9.4 (7.6–11.3)	0 (0–0)	8.7 (6.7–10.7)	3 (1.7–4.2)	13.7 (12.0–15.4)
Karnataka	0.8 (0–3)	11.3 (5.8–16.8)	1.7 (0.8–2.6)	11.8 (10.3–13.3)	2.6 (1.2–4.0)	12.5 (10.3–14.6)
Tamil Nadu	0 (0–0)	14.7 (11.4–18.0)	0 (0–0)	14.4 (12.7–16.0)	1.5 (0.7–2.2)	17 (15.1–18.9)

The IV Probit Models applied earlier for CHE25 were repeated for CHE40 and the pattern of the results remained similar (Additional file 6).

#### CHE10 incidence

CHE10 incidence was similar for the PFHI-enrolled and the non-insured. CHE10 incidence was several times greater for utilization in private sector (Additional file 7).

The IV Probit Models applied earlier were repeated for CHE10 and the pattern of the results remained similar (Additional file 8).

## Discussion

In the current study using robust IV approach, the utilisation (hospitalization) was not found to be associated with PFHI-enrollment in any of the three states. Hospitalisation was found to be more likely for older age groups, women and more educated persons, as reported by some other studies [24]. A recent study of three other states in India has reported no increase in hospital utilisation due to insurance [21]. Some studies have concluded that utilisation increased due to PFHI in India [8]. The mixed results could be due to differences in the methods applied, apart from the differences in populations, schemes and time-periods of different studies [8, 21, 22, 24].

The private sector dominated the overall empanelment of hospitals under PFHI as well as utilisation. Considering their lower availability as compared to the private hospitals under PFHI empanelment, lower utilization of public hospitals was on expected lines.

The current study taking into account the endogeneity of PFHI-enrollment showed that such insurance coverage had no relationship with OOPE or CHE in any of the three states. The inability of PFHI in ensuring financial protection for hospital-care is consistent with many other studies of PFHI in India [8, 18–28]. OOPE and CHE incidence in the current study was several times higher for private-sector hospitalizations irrespective of enrollment under PFHI, as found in earlier studies in India [24].

In Karnataka, an earlier study had found that PFHI resulted in reducing OOPE substantially for tertiary care [31]. The study was carried out in initial phase of VAS programme when it was being rolled out between 2010 and 2012. The matching applied in the study was inadequate for addressing endogeneity. The study had used OLS to estimate effect of insurance on OOPE, which can have severe limitations. A qualitative study had reported several problems in implementation of PFHI in Karnataka [46].

Fan et al. (2012) evaluated RAS programme in Andhra Pradesh during very early stages of its roll-out [9]. They analysed consumption expenditure data of NSS surveys between 1999 and 2008 using DID. The evidence used was indirect because the surveys did not have questions on insurance coverage, type of hospital used or type of illness etc. The study reported a small decline in in-patient OOPE in first phase of the roll-out but impact was not significant in the second phase. The study reported that the impact of PFHI was poor for most vulnerable social groups of Scheduled Castes and Scheduled Tribes. The impact of PFHI on Catastrophic Expenditure was not significant. A later study disagreed with the methods used by Fan et al. [41]. A study comparing Andhra Pradesh and Maharashtra between 2004 and 2012 suggested that OOPE had risen slower in Andhra Pradesh and using indirect evidence suggested that the PFHI in Andhra Pradesh was useful in reducing OOPE [30, 41]. Another study using the same dataset attributed the success of PFHI in Andhra Pradesh to involvement of private sector [42]. One study, also during early stages of RAS, used a different approach and dataset and showed that PFHI was ineffective in protecting the poor households from health related economic shocks [29]. The current study using direct evidence found that PFHI did not reduce OOPE in Andhra Pradesh and utilisation in private-sector remained equally expensive for the insurance-enrolled and the uninsured individuals. The current study is based on a survey done in 2014, when the three schemes were in place for more than 5 years. This was not the case for earlier evaluations of PFHI in Andhra Pradesh and Karnataka.

Why did OOPE remain high under PFHI? Some studies have found ‘double-billing’ by hospitals as a cause of OOPE under PFHI in India [26, 27]. ‘Double billing’ in the context of PFHI has been referred to the situation when hospitals, while claiming the amount for a service from insurance side, also charged illegal copayments from patients for the same service or asked them to buy drugs, diagnostics and consumables from outside. Tendencies to charge extra from the patients, despite PFHI cover have been reported from several states of India [26–28]. This is consistent with the explanation provided by Gertler and Solon that private providers in LMICs tend to appropriate the insurance benefit by charging extra from patients insured under PFHI [65]. According to them, PFHI in most LMICs could face similar challenges in financial protection because of poor ability to impose price-regulation on private providers [65]. Some studies have pointed out serious problems of moral hazard among private providers empanelled under PFHI in India [66–70]. According to them, there is poor regulation of private providers under the insurance schemes in India and they are able to influence government decision making and implementation. The findings of the current study in terms of persistent OOPE under PFHI may also be related to provider behavior and poor regulation. The relative size of OOPE in private and public sector hospitals, suggests that the share of the public sector could be increased in provisioning to bring down overall OOPE. The above suggestion will be valid where similar services are available from both sectors.

Studies have recommended that stronger supervision by state authorities, better mechanisms for addressing grievances and a 24-h helpline should be implemented to address the problem of ‘double-billing’ [18, 19, 26]. Another recommendation in literature is to improve awareness [19, 26]. In the current study, education level of individuals was positively associated with hospital-utilization but not with lower OOPE. Promotion of citizen ‘voice’ has been recommended as a mechanism to address gaps in healthcare [71]. However, international literature also points out that citizen ‘voice’ has received more emphasis in rhetoric of healthcare reforms than in actual practice [71]. The schemes in the current study did not seem to have citizen-committees or consultations. The schemes had a mechanism of ‘voice’ in form of formal complaint procedures and 24 h help lines. The persistent OOPE indicates that the mechanism though necessary, did not seem to be sufficient to control undesirable provider behavior.

Studies have suggested that the vertical cover of INR 30,000 annual assured sum per family might be insufficient, thereby causing possibility of CHE under RSBY [18, 22]. However, a study had shown that PFHI cover above INR 30,000 was not associated with lower OOPE [24]. The current study examined the three states which had the maximum vertical annual cover among all Indian states in 2014, around INR 200,000 per family. The current study suggests that increase in annual sum assured is unlikely to provide the desired financial protection.

A systematic review of PFHI in LMICs has reported that there is no evidence of impact of PFHI in reducing OOPE or improving financial protection, especially when endogeneity of insurance-enrollment was taken into account [6]. Some studies have shown positive impact of PFHI [52, 53]. In other countries, studies have reported increase in OOPE due to PFHI [34, 72, 73]. The literature often attributes the increase in OOPE to increased or un-necessary utilisation due to PFHI [7, 34]. In the current study, PFHI did not increase hospitalization rates in any of three states, yet OOPE did not decline for hospital-care. This further points to the possibility of ‘double-billing’ being a major cause of high OOPE under PFHI in India. An important attribute of PFHI schemes in India seems to be that they are publicly funded but mainly provided through for-profit private providers. This feature is common in PFHI schemes of some of the LMICs with mixed health systems, e.g. Morocco, Indonesia and Philippines. Similar problems like over-charging and poor control have been reported from such schemes [65, 74, 75].

In global literature, one of the gaps in purchasing is inadequate separation between provider and purchaser [76]. In current study, PFHIs had separated these roles to varying extents. In Tamil Nadu, there was an insurance company playing the role as purchaser and it was completely separate from the state government and linked only through a contract. In Andhra Pradesh and Karnataka, state governments created ‘Trusts’ to act as purchaser. A ‘Trust’ though incorporated as a separate body was still headed by state health officials and was under overall hierarchical control of state government. Thus, the ‘Insurance’ intermediary model seems to involve relatively greater separation of policy making, purchasing and providing roles, compared to the ‘Trust’ model. In India, there is a current debate regarding design of purchasing arrangements under PFHIs - whether to use a Trust or Insurance Company as purchaser [12, 77]. A study of Andhra Pradesh has suggested that Trust model offered some advantages, especially in reducing administrative costs. There was saving of some administrative cost under ‘Trust’ because no insurance firm was contracted to act as an intermediary. The study did not examine impact on OOPE but reported problem of formation of cartels by private hospitals [77]. We note that in the three states whose analysis was presented here, the experience is that PFHI schemes were unable to provide effective financial protection despite one opting for an Insurance model and two going for a Trust model and all three having a provider-purchaser split in place.

There is a recognition that governance and control needs to be strong for purchasing to be successful [74]. The states covered in the current study were known for having better governance and technical capacity. They were pioneers in starting PFHI programmes in India and were more experienced than other states. Studies using multiple parameters have consistently ranked them among top five or six states in India in terms of governance [78–80]. The above states showed considerable political support to PFHI schemes, cutting across party lines. The benefit stipulated in the PFHIs was of free cashless service covering pre and post operative care, diagnostics, drugs and transportation. The contracts forbade the hospitals from charging any copayments. Yet, the mechanism of contracting could not prevent private hospitals from taking extra money from patients, even in these states.

### Limitations

The NSS dataset does not distinguish between older insurance schemes of Central Government Health Services (CGHS) and Employee State Insurance (ESI) for the formally employed and the current wave of PFHIs that were the focus of this study. Other studies have reported that CGHS and ESIS form a very small proportion of PFHI enrollment and do not affect the results materially [20, 24].

## Conclusion

The study addresses the challenge of endogeneity when evaluating insurance and adds to understanding of insurance based health coverage in India. The three states studied here had better track record in governance and their PFHI schemes had substantially larger vertical cover among Indian states. Yet they failed to improve access or financial protection for hospitalization services for those enrolled under PFHI. This has important lessons for new policies in India and for the naïve assumption that an increase in sum assured would solve the problems of persistent OOPE and CHE despite insurance coverage. Nor is the creation of Trusts as purchasers, or provider-purchaser splits solving these problems. There is clearly a need for much tighter regulation and control. The study raises doubts regarding effectiveness of contracting under PFHIs to influence provider-behavior in Indian context. Further research is required to understand what could address the gaps that lead to poor financial protection outcomes under PFHI in India.

## Supplementary information

**Additional file 1.** Study Variables.

**Additional file 2.** Socio-Economic and Demographic Profile of Sample.

**Additional file 3.** IV Probit regression for Hospital Utilisation.

**Additional file 4.** 2sls regression for size of OOPE for hospitalization.

**Additional file 5.** IV PROBIT Regression for CHE25.

**Additional file 6.** IVPROBIT Regression for CHE40.

**Additional file 7.** Incidence of CHE10.

**Additional file 8.** IV PROBIT Regression for CHE10.

## Data Availability

The dataset used for analysis during the current study is available in the Ministry of Statistics and Programme Implementation, Government of India repository, [http://mospi.nic.in/national-sample-survey-office-nsso]. The datasets are available also at [microdata.gov.in/nada43/index.php/catalog/105] and [microdata.gov.in/nada43/index.php/catalog/135] for 2004 and 2014 rounds of NSS respectively.

## Abbreviations

2sls: Two Stage Least Squares
BPL: Below poverty line
CHE: Catastrophic health expenditure
CHE10: Catastrophic Health Expenditure computed using the threshold of 10% of usual annual consumption expenditure
CHE25: Catastrophic Health Expenditure computed using the threshold of 25% of usual annual consumption expenditure
CHE40: Catastrophic Health Expenditure computed using the threshold of 40% of usual annual consumption expenditure
CI: Confidence Interval
CMCHIS: Chief Minister’s Comprehensive Health Insurance Scheme
INR: Indian Rupee
IV: Instrumental Variable
LMIC: Low and Medium Income Countries
NSS: National sample survey
OLS: Ordinary Least Squares
OOPE: Out-of-pocket expenditure
PFHI: Public Funded Health Insurance
PMJAY: Pradhan Mantri Jan Arogaya Yojana
RAS: Rajiv Arogayasri Scheme
RSBY: Rashtriya Swasthya Bima Yojana
TNHSS: Tamil Nadu Health Systems Society
TPA: Third Party Administrator
UHC: Universal Health Coverage
USD: US Dollar
VAS: Vajpayee Arogyasri Scheme
