# Supplementary material for: Utilisation and financial protection for hospital care under publicly funded health insurance in three states in Southern India
Source: BMC Health Serv Res. 2019 Dec 27;19:1004. doi: 10.1186/s12913-019-4849-8 (PMC6935172; doi:10.1186/s12913-019-4849-8)
Supplement: Supplementary file 1 — Additional file 1. Study Variables. [file 12913_2019_4849_MOESM1_ESM.docx]

|  | **Additional file 1 - Study Variables** | |  |
| --- | --- | --- | --- |
|  |  |  |  |
| Sl | **Variable Name** | **Variable Description** | **Category** |
| 1 | sex | Sex of Individual | Male |
|  |  |  | Female |
| 2 | cat_edu | Education Category of Individual | Not Literate |
|  |  |  | Primary |
|  |  |  | Higher Secondary |
|  |  |  | Graduate or above |
| 3 | sgroup | Social Group of Individual | Scheduled Tribes |
|  |  |  | Scheduled Castes |
|  |  |  | Other Backward Classes |
|  |  |  | Others |
| 4 | quintile | Quintiles of Consumption Expenditure of Individual's Household | Poorest |
|  |  |  | Poor |
|  |  |  | Middle |
|  |  |  | Rich |
|  |  |  | Richest |
| 5 | cat_age | Age Category of Individual | <1 year |
|  |  |  | 1-4 Years |
|  |  |  | 5-14 Years |
|  |  |  | 15-48 Years |
|  |  |  | 49-59 Years |
|  |  |  | 60 Years and above |
| 6 | place | Place of Residence of Individual | Rural |
|  |  |  | Urban |
| 7 | hospitalised_yes | Hospitalisation episode in last one year | Yes |
|  |  |  | No |
| 8 | govt_insurance | Whether individual enrolled under PFHI scheme | Yes |
|  |  |  | No |
| 9 | type_hospital | Type of Hospital in which hospitalisation took place | Public Hospital |
|  |  |  | Private Hospital |
| 10 | Cat_disease | Category of Disease/Condition for which hospitalisation episodetook place | Communicable Diseases |
|  |  |  | Non Communicable Diseases |
|  |  |  | Maternal |
|  |  |  | Emergency & Injury |
|  |  |  | Others |
| 11 | hospital_duration | Duration of Hospitalisation Episode | <= 3 days |
|  |  |  | > 3 days |
| 12 | oope_final | Out of Pocket Expenditure (OOPE) |  |
| 13 | CHE10 | Catastrophic Health Expenditure at 10% threshold (OOPE > 10% of Annual Consumption Expenditure of concerned Household) | Yes |
|  |  |  | No |
| 14 | CHE25 | Catastrophic Health Expenditure at 25% threshold (OOPE > 25% of Annual Consumption Expenditure of concerned Household) | Yes |
|  |  |  | No |
| 15 | CHE40 | Catastrophic Health Expenditure at 40% threshold (OOPE > 40% of Annual Consumption Expenditure of concerned Household) | Yes |
|  |  |  | No |
| 16 | Year | Year of Survey | 2004 |
|  |  |  | 2014 |
