# Supplementary material for: Utilisation and financial protection for hospital care under publicly funded health insurance in three states in Southern India
Source: BMC Health Serv Res. 2019 Dec 27;19:1004. doi: 10.1186/s12913-019-4849-8 (PMC6935172; doi:10.1186/s12913-019-4849-8)
Supplement: Supplementary file 2 — Additional file 2. Socio-Economic and Demographic Profile of Sample. [file 12913_2019_4849_MOESM2_ESM.docx]

| **A2: Additional File 2: Socio-Economic and Demographic Profile of Sample** | | | | | | | | | | | | |  |
| --- | --- | --- | --- | --- | --- | --- | --- | --- | --- | --- | --- | --- | --- |
|  |  | **Andhra Pradesh** | | | | **Karnataka** | | | | **Tamil Nadu** | | | |
|  |  | **2004** | | **2014** | | **2004** | | **2014** | | **2004** | | **2014** | |
| **Variable** | **Category** | **Frequency** | **%** | **Frequency** | **%** | **Frequency** | **%** | **Frequency** | **%** | **Frequency** | **%** | **Frequency** | **%** |
| Sex | Male | 11,188 | 50 | 5,301 | 49.8 | 8,519 | 50.2 | 7,530 | 51.1 | 10,571 | 50 | 7,956 | 49 |
|  | Female | 11,199 | 50 | 5,335 | 50.2 | 8,467 | 49.8 | 7,197 | 48.9 | 10,723 | 50 | 8,134 | 51 |
| Education | Not Literate | 14,127 | 63 | 4,069 | 38.2 | 9,365 | 55.1 | 4,430 | 30.1 | 10,413 | 49 | 4,106 | 26 |
|  | Primary | 2,657 | 12 | 2,878 | 27.1 | 2,265 | 13.3 | 3,778 | 25.7 | 3,938 | 18 | 4,708 | 29 |
|  | Higher Secondary | 4,010 | 18 | 2,996 | 28.2 | 4,000 | 23.6 | 5,525 | 37.5 | 4,890 | 23 | 5,884 | 37 |
|  | Graduate or above | 1,586 | 7.1 | 693 | 6.5 | 1,356 | 7.98 | 995 | 6.75 | 2,052 | 9.6 | 1,392 | 8.7 |
| Social Group | Scheduled Tribes | 1,319 | 5.9 | 683 | 6.4 | 1,371 | 8.07 | 966 | 6.56 | 357 | 1.7 | 374 | 2.3 |
|  | Scheduled Castes | 4,569 | 20 | 2,066 | 19.4 | 2,901 | 17.1 | 2,248 | 15.3 | 5,245 | 25 | 3,658 | 23 |
|  | Other Backward Classes | 10,609 | 47 | 4,771 | 44.9 | 5,494 | 32.4 | 6,788 | 46.1 | 14,621 | 69 | 11,748 | 73 |
|  | Others | 5,890 | 26 | 3,117 | 29.3 | 7,220 | 42.5 | 4,725 | 32.1 | 1,071 | 5 | 310 | 1.9 |
| Quintiles of Consumption Expenditure | Poorest | 6,333 | 28 | 722 | 6.8 | 3,454 | 20.3 | 1,371 | 9.31 | 5,837 | 27 | 1,529 | 9.5 |
|  | Poor | 5,510 | 25 | 1,728 | 16.3 | 3,812 | 22.4 | 3,360 | 22.8 | 4,897 | 23 | 2,573 | 16 |
|  | Middle | 4,233 | 19 | 3,317 | 31.2 | 3,498 | 20.6 | 3,482 | 23.7 | 4,133 | 19 | 4,075 | 25 |
|  | Rich | 3,217 | 14 | 2,808 | 26.4 | 3,185 | 18.8 | 3,617 | 24.6 | 3,320 | 16 | 4,578 | 28 |
|  | Richest | 3,094 | 14 | 2,062 | 19.4 | 3,038 | 17.9 | 2,896 | 19.7 | 3,107 | 15 | 3,335 | 21 |
| Age | <1 year | 314 | 1.4 | 149 | 1.4 | 231 | 1.38 | 225 | 1.55 | 307 | 1.5 | 198 | 1.3 |
|  | 1-4 Years | 1,718 | 7.7 | 659 | 6.3 | 1,319 | 7.84 | 1,001 | 6.88 | 1,522 | 7.2 | 927 | 5.8 |
|  | 5-14 Years | 4,959 | 22 | 1,773 | 16.9 | 3,635 | 21.6 | 2,500 | 17.2 | 4,191 | 20 | 2,519 | 16 |
|  | 15-48 Years | 11,939 | 54 | 6,120 | 58.2 | 9,094 | 54.1 | 8,317 | 57.2 | 11,381 | 54 | 8,679 | 55 |
|  | 49-59 Years | 1,853 | 8.3 | 1,010 | 9.6 | 1,553 | 9.24 | 1,387 | 9.53 | 2,056 | 9.7 | 1,944 | 12 |
|  | 60 Years and above | 1,424 | 6.4 | 803 | 7.6 | 981 | 5.84 | 1,123 | 7.72 | 1,650 | 7.8 | 1,609 | 10 |
| Place | Rural | 16,224 | 72 | 7,400 | 69.6 | 11,990 | 70.6 | 9,122 | 61.9 | 13,808 | 65 | 8,124 | 50 |
|  | Urban | 6,163 | 28 | 3,236 | 30.4 | 4,996 | 29.4 | 5,605 | 38.1 | 7,486 | 35 | 7,966 | 50 |
| Hospitalised in last one year | Yes | 513 | 2.3 | 593 | 5.6 | 380 | 2.23 | 726 | 4.93 | 763 | 3.6 | 914 | 5.7 |
|  | No | 21,874 | 98 | 10,043 | 94.4 | 16,606 | 97.8 | 14,001 | 95.1 | 20,531 | 96 | 15,176 | 94 |
| Enrolled under PFHI scheme | Yes | 0 | 0 | 6,655 | 62.6 | 0 | 0 | 765 | 5.19 | 0 | 0 | 2,858 | 18 |
|  | No | 22,387 | 100 | 3,981 | 37.4 | 16,986 | 100 | 13,962 | 94.8 | 21,294 | 100 | 13,232 | 82 |
