# Supplementary material for: Utilisation and financial protection for hospital care under publicly funded health insurance in three states in Southern India
Source: BMC Health Serv Res. 2019 Dec 27;19:1004. doi: 10.1186/s12913-019-4849-8 (PMC6935172; doi:10.1186/s12913-019-4849-8)
Supplement: Supplementary file 3 — Additional file 3. IV Probit regression for Hospital Utilisation. [file 12913_2019_4849_MOESM3_ESM.docx]

**Additional file 3 - IV Probit regression for Hospital Utilisation**

**Table S3.1 - IV Probit regression for Hospital Utilisation – Andhra Pradesh**

| Two-step Probit with endogenous regressors (IV: Social Group) Number of obs= 32,713 | | | | | | | | | | | | |
| --- | --- | --- | --- | --- | --- | --- | --- | --- | --- | --- | --- | --- |
| Variable | | Category | | | Coef. | Std. Err. | | z | P>z | | [95% Conf.Interval] | |
| Government insurance (Instrumented) | | Yes | | | -0.085 | 0.526 | | -0.160 | 0.872 | | -1.116 | 0.947 |
| Education | | Not Literate | | | 0 |  | |  |  | |  |  |
|  |  | Primary | | | 0.042 | 0.030 | | 1.410 | 0.157 | | -0.016 | 0.101 |
|  |  | Higher Secondary | | | 0.016 | 0.027 | | 0.590 | 0.555 | | -0.037 | 0.069 |
|  |  | Graduate or Above | | | -0.027 | 0.049 | | -0.550 | 0.583 | | -0.122 | 0.069 |
| Quintile | | Poorest | | | 0 |  | |  |  | |  |  |
|  |  | Poor | | | 0.001 | 0.033 | | 0.040 | 0.970 | | -0.063 | 0.066 |
|  |  | Middle | | | 0.010 | 0.038 | | 0.260 | 0.797 | | -0.065 | 0.084 |
|  |  | Rich | | | -0.023 | 0.042 | | -0.550 | 0.582 | | -0.106 | 0.060 |
|  |  | Richest | | | -0.049 | 0.038 | | -1.300 | 0.193 | | -0.123 | 0.025 |
| Age | | <1 year | | | 0 |  | |  |  | |  |  |
|  |  | 1-4 Years | | | -0.074 | 0.106 | | -0.700 | 0.482 | | -0.282 | 0.133 |
|  |  | 5-14 Years | | | -0.243 | 0.131 | | -1.850 | 0.065 | | -0.500 | 0.015 |
|  |  | 15-48 Years | | | 0.252 | 0.140 | | 1.800 | 0.072 | | -0.022 | 0.527 |
|  |  | 49-59 Years | | | 0.488 | 0.140 | | 3.480 | 0.000 | | 0.213 | 0.762 |
|  |  | 60 Years and above | | | 0.586 | 0.136 | | 4.300 | 0.000 | | 0.319 | 0.853 |
| Sex | | Male | | | 0 |  | |  |  | |  |  |
|  |  | Female | | | 0.067 | 0.020 | | 3.380 | 0.001 | | 0.028 | 0.107 |
| Place | | Rural | | | 0 |  | |  |  | |  |  |
|  |  | Urban | | | 0.031 | 0.041 | | 0.750 | 0.454 | | -0.049 | 0.111 |
| Year | | 2014 | | | 0 |  | |  |  | |  |  |
|  |  | 2004 | | | -0.476 | 0.298 | | -1.600 | 0.109 | | -1.060 | 0.107 |
|  | | _cons | | | -1.105 | 0.195 | | -5.660 | 0.000 | | -1.488 | -0.723 |
|  | | Wald test of exogeneity: chi2(1) = 0.01 Prob> chi2 = 0.9049 | | | | | | | | | | |
| Weak Instruments Robust Tests for IV Probit | | | | | | | | | |  |  |  |
| Test | Statistic | | p-value | Conf. level | | | Conf.Set | | |  |  |  |
| CLR | stat(.) =0.03 | | 0.870 | 95% | | | [-1.14762,.978179] | | |  |  |  |
| K | chi2(1) =0.03 | | 0.873 | 95% | | | [-1.14762,.978179] | | |  |  |  |
| J | chi2(2) =3.08 | | 0.215 | 95% | | | entire grid | | |  |  |  |
| K-J | NA | | 0.890 | 95% (96%,99%) | | | [ -1.1893,1.01986] | | |  |  |  |
| AR | chi2(3) =3.1 | | 0.376 | 95% | | | [-1.23098,1.06154] | | |  |  |  |
| Wald | chi2(1) =0.03 | | 0.872 | 95% | | | [-1.11635,.946917] | | |  |  |  |

**Table S3.2: IV Probit regression for Hospital Utilisation – Karnataka**

| Two-step Probit with endogenous regressors (IV: Social Group) Number of obs= 31,366 | | | | | | | | | | | | |
| --- | --- | --- | --- | --- | --- | --- | --- | --- | --- | --- | --- | --- |
| Variable | | Category | | | Coef. | | Std. Err. | z | P>z | | [95% Conf.Interval] | |
| Government insurance (Instrumented) | | Yes | | | 1.378 | | 1.336 | 1.030 | 0.303 | | -1.242 | 3.997 |
| Education | | Not Literate | | | 0 | |  |  |  | |  |  |
|  |  | Primary | | | 0.091 | | 0.030 | 3.060 | 0.002 | | 0.033 | 0.150 |
|  |  | Higher Secondary | | | 0.057 | | 0.032 | 1.790 | 0.074 | | -0.006 | 0.119 |
|  |  | Graduate or Above | | | 0.106 | | 0.041 | 2.580 | 0.010 | | 0.025 | 0.187 |
| Quintile | | Poorest | | | 0 | |  |  |  | |  |  |
|  |  | Poor | | | -0.134 | | 0.038 | -3.510 | 0.000 | | -0.208 | -0.059 |
|  |  | Middle | | | -0.133 | | 0.037 | -3.620 | 0.000 | | -0.205 | -0.061 |
|  |  | Rich | | | -0.204 | | 0.036 | -5.600 | 0.000 | | -0.276 | -0.133 |
|  |  | Richest | | | -0.245 | | 0.039 | -6.320 | 0.000 | | -0.322 | -0.169 |
| Age | | <1 year | | | 0 | |  |  |  | |  |  |
|  |  | 1-4 Years | | | 0.130 | | 0.072 | 1.800 | 0.071 | | -0.011 | 0.271 |
|  |  | 5-14 Years | | | -0.110 | | 0.071 | -1.560 | 0.120 | | -0.249 | 0.029 |
|  |  | 15-48 Years | | | 0.384 | | 0.066 | 5.860 | 0.000 | | 0.256 | 0.513 |
|  |  | 49-59 Years | | | 0.513 | | 0.073 | 7.060 | 0.000 | | 0.371 | 0.656 |
|  |  | 60 Years and above | | | 0.761 | | 0.071 | 10.770 | 0.000 | | 0.623 | 0.900 |
| Sex | | Male | | | 0 | |  |  |  | |  |  |
|  |  | Female | | | 0.190 | | 0.020 | 9.630 | 0.000 | | 0.151 | 0.228 |
| Place | | Rural | | | 0 | |  |  |  | |  |  |
|  |  | Urban | | | 0.041 | | 0.023 | 1.800 | 0.072 | | -0.004 | 0.086 |
| Year | | 2014 | | | 0 | |  |  |  | |  |  |
|  |  | 2004 | | | -0.354 | | 0.069 | -5.160 | 0.000 | | -0.489 | -0.220 |
|  | | _cons | | | -1.392 | | 0.082 | -17.060 | 0.000 | | -1.552 | -1.232 |
|  | | Wald test of exogeneity: chi2(1) = 0.81 Prob> chi2 = 0.3688 | | | | | | | | | |  |
| Weak Instruments Robust Tests for IV Probit | | | | | | | | | | | |  |
| Test | Statistic | | p-value | Conf. level | | Conf.Set | | | |  |  |  |
| CLR | stat(.) =1.1 | | 0.300 | 95% | | [-1.21505,4.18264] | | | |  |  |  |
| K | chi2(1) =1.06 | | 0.302 | 95% | | [-1.21505,4.28848] | | | |  |  |  |
| J | chi2(2) =2.67 | | 0.263 | 95% | | entire grid | | | |  |  |  |
| K-J |  | | 0.355 | 95% (96%,99%) | | [-1.42672,4.39432] | | | |  |  |  |
| AR | chi2(3) =3.73 | | 0.292 | 95% | | [ -1.6384,4.71183] | | | |  |  |  |
| Wald | chi2(1) =1.06 | | 0.303 | 95% | | [-1.24151,3.99743] | | | |  |  |  |

**Table S3.3: IV Probit for Hospital Utilisation – Tamil Nadu**

| Two-step Probit with endogenous regressors (IV : Social Group) Number of obs= 36,982 | | | | | | | | | | | | |
| --- | --- | --- | --- | --- | --- | --- | --- | --- | --- | --- | --- | --- |
| Variable | | Category | | | Coef. | | Std. Err. | z | P>z | | [95% Conf.Interval] | |
| Government insurance (Instrumented) | | Yes | | | -0.130 | | 1.398 | -0.090 | 0.926 | | -2.871 | 2.611 |
| Education | | Not Literate | | | 0 | |  |  |  | |  |  |
|  |  | Primary | | | 0.059 | | 0.025 | 2.320 | 0.020 | | 0.009 | 0.109 |
|  |  | Higher Secondary | | | 0.040 | | 0.024 | 1.630 | 0.103 | | -0.008 | 0.088 |
|  |  | Graduate or Above | | | 0.048 | | 0.034 | 1.430 | 0.152 | | -0.018 | 0.114 |
| Quintile | | Poorest | | | 0 | |  |  |  | |  |  |
|  |  | Poor | | | -0.067 | | 0.033 | -2.040 | 0.041 | | -0.131 | -0.003 |
|  |  | Middle | | | -0.092 | | 0.043 | -2.130 | 0.033 | | -0.176 | -0.007 |
|  |  | Rich | | | -0.102 | | 0.041 | -2.500 | 0.012 | | -0.181 | -0.022 |
|  |  | Richest | | | -0.110 | | 0.054 | -2.030 | 0.042 | | -0.217 | -0.004 |
| Age | | <1 year | | | 0 | |  |  |  | |  |  |
|  |  | 1-4 Years | | | 0.191 | | 0.091 | 2.110 | 0.035 | | 0.013 | 0.370 |
|  |  | 5-14 Years | | | -0.047 | | 0.108 | -0.440 | 0.660 | | -0.258 | 0.163 |
|  |  | 15-48 Years | | | 0.521 | | 0.110 | 4.750 | 0.000 | | 0.306 | 0.737 |
|  |  | 49-59 Years | | | 0.760 | | 0.128 | 5.930 | 0.000 | | 0.508 | 1.011 |
|  |  | 60 Years and above | | | 0.895 | | 0.121 | 7.390 | 0.000 | | 0.658 | 1.133 |
| Sex | | Male | | | 0 | |  |  |  | |  |  |
|  |  | Female | | | 0.167 | | 0.017 | 9.790 | 0.000 | | 0.133 | 0.200 |
| Place | | Rural | | | 0 | |  |  |  | |  |  |
|  |  | Urban | | | 0.006 | | 0.034 | 0.170 | 0.862 | | -0.060 | 0.072 |
| Year | | 2014 | | | 0 | |  |  |  | |  |  |
|  |  | 2004 | | | -0.452 | | 0.239 | -1.890 | 0.059 | | -0.921 | 0.017 |
|  | | _cons | | | -1.363 | | 0.154 | -8.840 | 0.000 | | -1.665 | -1.060 |
|  | | Wald test of exogeneity: chi2(1) = 0.00 Prob> chi2 = 0.9471 | | | | | | | | | |  |
| Weak Instruments Robust Tests for IV Probit | | | | | | | | | | | |  |
| Test | Statistic | | p-value | Conf. level | | Conf.Set | | | |  |  |  |
| CLR | stat(.) =0.01 | | 0.927 | 95% | | [-3.61873,3.24747] | | | |  |  |  |
| K | chi2(1) =0.01 | | 0.927 | 95% | | [-3.72947,3.35822] | | | |  |  |  |
| J | chi2(2) =2.68 | | 0.262 | 95% | | entire grid | | | |  |  |  |
| K-J |  | | 0.944 | 95% (96%,99%) | | [-3.95097,3.69045] | | | |  |  |  |
| AR | chi2(3) =2.69 | | 0.442 | 95% | | [-4.06171,3.69045] | | | |  |  |  |
| Wald | chi2(1) =0.01 | | 0.926 | 95% | | [ -2.8712,2.61069] | | | |  |  |  |
