# Supplementary material for: Utilisation and financial protection for hospital care under publicly funded health insurance in three states in Southern India
Source: BMC Health Serv Res. 2019 Dec 27;19:1004. doi: 10.1186/s12913-019-4849-8 (PMC6935172; doi:10.1186/s12913-019-4849-8)
Supplement: Supplementary file 4 — Additional file 4. 2sls regression for size of OOPE for hospitalization. [file 12913_2019_4849_MOESM4_ESM.docx]

**Additional file 4: 2sls regression for size of OOPE for hospitalization**

**Table S4.1 - 2sls regression for size of OOPE for hospitalization – Andhra Pradesh**

| Instrumental variables (2sls) regression (IV: Sex) Number of obs= 4,520 | | | | | | | |
| --- | --- | --- | --- | --- | --- | --- | --- |
| Variable | Category | Coef. | Std. Err. | z | P>z | [95% Conf.Interval] | |
| Government insurance (Instrumented) | Yes | 2944.541 | 35372.290 | 0.080 | 0.934 | -66383.880 | 72272.960 |
| Education | Not Literate | 0 |  |  |  |  |  |
|  | Primary | 1336.842 | 1261.596 | 1.060 | 0.289 | -1135.841 | 3809.525 |
|  | Higher Secondary | 3799.487 | 2460.188 | 1.540 | 0.122 | -1022.394 | 8621.368 |
|  | Graduate or Above | 7327.140 | 6184.032 | 1.180 | 0.236 | -4793.341 | 19447.620 |
| Quintile | Poorest | 0 |  |  |  |  |  |
|  | Poor | 549.440 | 1964.762 | 0.280 | 0.780 | -3301.423 | 4400.303 |
|  | Middle | 3548.715 | 3274.627 | 1.080 | 0.278 | -2869.436 | 9966.867 |
|  | Rich | 3958.840 | 3419.947 | 1.160 | 0.247 | -2744.133 | 10661.810 |
|  | Richest | 11880.700 | 2543.662 | 4.670 | 0.000 | 6895.210 | 16866.180 |
| Age | <1 year | 0 |  |  |  |  |  |
|  | 1-4 Years | -2723.695 | 5558.203 | -0.490 | 0.624 | -13617.570 | 8170.183 |
|  | 5-14 Years | -1361.793 | 8949.464 | -0.150 | 0.879 | -18902.420 | 16178.830 |
|  | 15-48 Years | 1950.890 | 10221.150 | 0.190 | 0.849 | -18082.190 | 21983.970 |
|  | 49-59 Years | 1476.420 | 10398.090 | 0.140 | 0.887 | -18903.460 | 21856.300 |
|  | 60 Years and above | 2532.100 | 9666.187 | 0.260 | 0.793 | -16413.280 | 21477.480 |
| Social group | ST | 0 |  |  |  |  |  |
|  | SC | -786.974 | 3986.974 | -0.200 | 0.844 | -8601.300 | 7027.351 |
|  | OBC | -131.444 | 3879.357 | -0.030 | 0.973 | -7734.844 | 7471.956 |
|  | Others | 2813.050 | 4979.342 | 0.560 | 0.572 | -6946.280 | 12572.380 |
| Place | Rural | 0 |  |  |  |  |  |
|  | Urban | -1313.705 | 2654.099 | -0.490 | 0.621 | -6515.642 | 3888.233 |
| Year | 2014 | 0.000 |  |  |  |  |  |
|  | 2004 | 4357.597 | 21757.110 | 0.200 | 0.841 | -38285.550 | 47000.740 |
| Category of disease | Communicable | 0 |  |  |  |  |  |
|  | NCD | 6668.893 | 1275.668 | 5.230 | 0.000 | 4168.629 | 9169.156 |
|  | Maternal | -863.438 | 2868.653 | -0.300 | 0.763 | -6485.895 | 4759.019 |
|  | Emergency & Injury | 9230.221 | 1704.238 | 5.420 | 0.000 | 5889.975 | 12570.470 |
|  | Others | 2271.346 | 1435.434 | 1.580 | 0.114 | -542.053 | 5084.744 |
| Type of hospital | Public hospital | 0 |  |  |  |  |  |
|  | Private Hospital | 12400.040 | 1672.309 | 7.410 | 0.000 | 9122.376 | 15677.710 |
| Hospital duration | Less than 3 days | 0 |  |  |  |  |  |
|  | more than 3 days | 12180.900 | 1426.469 | 8.540 | 0.000 | 9385.069 | 14976.730 |
|  | _cons | -15560.240 | 16584.930 | -0.940 | 0.348 | -48066.110 | 16945.620 |
|  | Test of Endogeneity (. estat endog) | | | |  |  |  |
|  | Durbin (score) chi2(1) = .055782 (p = 0.8133) | | | |  |  |  |
|  | Wu-HausmanF(1,4494) = .055461 (p = 0.8138) | | | | |  |  |
|  | Test of Overidentifying Restrictions (. estat overid) | | | |  |  |  |
|  | No overidentifying restrictions | |  |  |  |  |  |

**Table S4.2: 2sls regression for size of OOPE for hospitalization – Karnataka**

| Instrumental variables (2sls) regression (IV: Social Group) Number of obs= 4,107 | | | | | | | | |
| --- | --- | --- | --- | --- | --- | --- | --- | --- |
| Variable | Category | Coef. | Std. Err. | z | | P>z | [95% Conf.Interval] | |
| Government insurance (Instrumented) | Yes | 45744.550 | 34789.840 | 1.310 | | 0.189 | -22442.280 | 113931.400 |
| Education | Not Literate | 0 |  |  | |  |  |  |
|  | Primary | 3508.340 | 1222.246 | 2.870 | | 0.004 | 1112.782 | 5903.898 |
|  | Higher Secondary | 4134.462 | 1487.854 | 2.780 | | 0.005 | 1218.322 | 7050.602 |
|  | Graduate or Above | 7713.850 | 2085.701 | 3.700 | | 0.000 | 3625.952 | 11801.750 |
| Quintile | Poorest | 0 |  |  | |  |  |  |
|  | Poor | -288.916 | 1644.754 | -0.180 | | 0.861 | -3512.574 | 2934.742 |
|  | Middle | 593.251 | 1647.079 | 0.360 | | 0.719 | -2634.965 | 3821.467 |
|  | Rich | -29.286 | 1925.052 | -0.020 | | 0.988 | -3802.319 | 3743.747 |
|  | Richest | 3676.937 | 2292.518 | 1.600 | | 0.109 | -816.316 | 8170.189 |
| Age | <1 year | 0 |  |  | |  |  |  |
|  | 1-4 Years | -3696.510 | 3841.881 | -0.960 | | 0.336 | -11226.460 | 3833.438 |
|  | 5-14 Years | -5962.432 | 3850.101 | -1.550 | | 0.121 | -13508.490 | 1583.627 |
|  | 15-48 Years | -4533.768 | 3564.573 | -1.270 | | 0.203 | -11520.200 | 2452.667 |
|  | 49-59 Years | -3848.031 | 3995.574 | -0.960 | | 0.336 | -11679.210 | 3983.150 |
|  | 60 Years and above | 164.907 | 3688.014 | 0.040 | | 0.964 | -7063.469 | 7393.282 |
| Sex | Male | 0 |  |  | |  |  |  |
|  | Female | -1130.525 | 1012.631 | -1.120 | | 0.264 | -3115.244 | 854.195 |
| Place | Rural | 0.000 |  |  | |  |  |  |
|  | Urban | -496.401 | 1021.189 | -0.490 | | 0.627 | -2497.894 | 1505.093 |
| Year | 2014 | 0 |  |  | |  |  |  |
|  | 2004 | 3327.877 | 2560.261 | 1.300 | | 0.194 | -1690.142 | 8345.897 |
| Category of disease | Communicable | 0 |  |  | |  |  |  |
|  | NCD | 10005.160 | 1347.084 | 7.430 | | 0.000 | 7364.927 | 12645.400 |
|  | Maternal | 5673.192 | 1518.479 | 3.740 | | 0.000 | 2697.027 | 8649.357 |
|  | Emergency & Injury | 15341.390 | 1910.511 | 8.030 | | 0.000 | 11596.860 | 19085.920 |
|  | Others | 3493.808 | 1433.158 | 2.440 | | 0.015 | 684.870 | 6302.745 |
| Type of hospital | Public hospital | 0 |  |  | |  |  |  |
|  | Private Hospital | 13226.610 | 1279.844 | 10.330 | | 0.000 | 10718.160 | 15735.060 |
| Hospital duration | Less than 3 days | 0 |  |  | |  |  |  |
|  | more than 3 days | 10950.040 | 980.399 | 11.170 | | 0.000 | 9028.492 | 12871.580 |
|  | _cons | -9334.300 | 3795.465 | -2.460 | | 0.014 | -16773.280 | -1895.325 |
|  | Test of Endogeneity (. estat endog) | | | | |  |  |  |
|  | Durbin (score) chi2(1) = 2.35464 (p = 0.1249) | | | | |  |  |  |
|  | Wu-HausmanF(1,4083) = 2.34223 (p = 0.1260) | | | | |  |  |  |
|  | Test of Overidentifying Restrictions(. estat overid) | | | | |  |  |  |
|  | Sargan (score) chi2(2) = 1.06166 (p = 0.5881) | | | |  |  |  |  |
|  | Basmann chi2(2) = 1.05547 (p = 0.5899) | | | |  |  |  |  |

**Table S4.3: 2sls regression for size of OOPE for hospitalization – Tamil Nadu**

| Instrumental variables (2sls) regression (IV: Social Group) Number of obs= 5,933 | | | | | | | |
| --- | --- | --- | --- | --- | --- | --- | --- |
| Variable | Category | Coef. | Std. Err. | z | P>z | [95% Conf.Interval] | |
| Government insurance (Instrumented) | Yes | 63942.380 | 49332.880 | 1.300 | 0.195 | -32748.280 | 160633.000 |
| Education | Not Literate | 0 |  |  |  |  |  |
|  | Primary | 1178.695 | 1779.413 | 0.660 | 0.508 | -2308.891 | 4666.281 |
|  | Higher Secondary | 5131.243 | 2318.064 | 2.210 | 0.027 | 587.920 | 9674.566 |
|  | Graduate or Above | 19263.450 | 2850.291 | 6.760 | 0.000 | 13676.980 | 24849.910 |
| Quintile | Poorest | 0 |  |  |  |  |  |
|  | Poor | 6448.362 | 2369.910 | 2.720 | 0.007 | 1803.424 | 11093.300 |
|  | Middle | 6011.584 | 2463.461 | 2.440 | 0.015 | 1183.290 | 10839.880 |
|  | Rich | 3947.432 | 2461.756 | 1.600 | 0.109 | -877.521 | 8772.384 |
|  | Richest | 11994.650 | 3162.298 | 3.790 | 0.000 | 5796.664 | 18192.640 |
| Age | <1 year | 0 |  |  |  |  |  |
|  | 1-4 Years | 611.805 | 5954.464 | 0.100 | 0.918 | -11058.730 | 12282.340 |
|  | 5-14 Years | -1470.319 | 5731.148 | -0.260 | 0.798 | -12703.160 | 9762.525 |
|  | 15-48 Years | -1180.879 | 5272.309 | -0.220 | 0.823 | -11514.410 | 9152.656 |
|  | 49-59 Years | 199.881 | 6054.509 | 0.030 | 0.974 | -11666.740 | 12066.500 |
|  | 60 Years and above | 4249.709 | 5231.279 | 0.810 | 0.417 | -6003.409 | 14502.830 |
| Sex | Male | 0 |  |  |  |  |  |
|  | Female | -1059.465 | 1423.336 | -0.740 | 0.457 | -3849.153 | 1730.223 |
| Place | Rural | 0 |  |  |  |  |  |
|  | Urban | 2255.265 | 1972.464 | 1.140 | 0.253 | -1610.693 | 6121.223 |
| Year | 2014 | 0 |  |  |  |  |  |
|  | 2004 | 16415.010 | 9519.505 | 1.720 | 0.085 | -2242.878 | 35072.900 |
| Category of disease | Communicable | 0 |  |  |  |  |  |
|  | NCD | 11516.590 | 2182.650 | 5.280 | 0.000 | 7238.676 | 15794.510 |
|  | Maternal | 5453.986 | 2969.163 | 1.840 | 0.066 | -365.466 | 11273.440 |
|  | Emergency & Injury | 10954.600 | 2599.201 | 4.210 | 0.000 | 5860.263 | 16048.940 |
|  | Others | 2606.089 | 2140.216 | 1.220 | 0.223 | -1588.657 | 6800.835 |
| Type of hospital | Public hospital | 0 |  |  |  |  |  |
|  | Private Hospital | 19130.050 | 1857.727 | 10.300 | 0.000 | 15488.970 | 22771.130 |
| Hospital duration | Less than 3 days | 0 |  |  |  |  |  |
|  | more than 3 days | 13550.060 | 1500.456 | 9.030 | 0.000 | 10609.220 | 16490.900 |
|  | _cons | -33010.930 | 8801.815 | -3.750 | 0.000 | -50262.170 | -15759.690 |
|  | Test of Endogeneity (. estat endog) | | | |  |  |  |
|  | Durbin (score) chi2(1) = 2.187 (p = 0.1392) | | | |  |  |  |
|  | Wu-HausmanF(1,5909) = 2.17896 (p = 0.1400) | | | |  |  |  |
|  | Test of Overidentifying Restrictions(. estat overid) | | | |  |  |  |
|  | Sargan (score) chi2(2) = .443373 (p = 0.8012) | | | |  |  |  |
|  | Basmann chi2(2) = .441537 (p = 0.8019) | | | |  |  |  |
