# Supplementary material for: Utilisation and financial protection for hospital care under publicly funded health insurance in three states in Southern India
Source: BMC Health Serv Res. 2019 Dec 27;19:1004. doi: 10.1186/s12913-019-4849-8 (PMC6935172; doi:10.1186/s12913-019-4849-8)
Supplement: Supplementary file 5 — Additional file 5. IV PROBIT Regression for CHE25. [file 12913_2019_4849_MOESM5_ESM.docx]

| **Additional file 5 - IV PROBIT Regression for CHE25** | | | | | | | | | | | | | | |  | |  | |  | |  |
| --- | --- | --- | --- | --- | --- | --- | --- | --- | --- | --- | --- | --- | --- | --- | --- | --- | --- | --- | --- | --- | --- |
|  |  |  |  | |  |  | |  | |  | |  | |  | |  | |  | |  | |
| **Table S5.1: IV PROBIT regression to find predictors of CHE25 – Andhra Pradesh** | | | | | | | | | | | | | | | | | | | | |  |
| Two-step Probit with endogenous regressors (IV: Place) Number of obs= 4,520 | | | | | | | | | | | | | | | | | | | | |  |
| Variable | | Category | | Coef. | | | | | Std. Err. | | Z | | | | P>z | | [95% Conf.Interval] | | | |  |
| Government insurance (Instrumented) | | Yes | | 1.407 | | | | | 0.881 | | 1.6 | | | | 0.11 | | -0.319 | | 3.134 | |  |
| Quintile | | Poorest | | 0 | | | | |  | |  | | | |  | |  | |  | |  |
|  |  | Poor | | -0.403 | | | | | 0.088 | | -4.6 | | | | 0 | | -0.574 | | -0.231 | |  |
|  |  | Middle | | -0.634 | | | | | 0.106 | | -5.96 | | | | 0 | | -0.843 | | -0.425 | |  |
|  |  | Rich | | -1.02 | | | | | 0.109 | | -9.33 | | | | 0 | | -1.235 | | -0.806 | |  |
|  |  | Richest | | -1.141 | | | | | 0.1 | | -11.43 | | | | 0 | | -1.337 | | -0.946 | |  |
| Age | | <1 year | | 0 | | | | |  | |  | | | |  | |  | |  | |  |
|  |  | 1-4 Years | | -0.226 | | | | | 0.25 | | -0.91 | | | | 0.365 | | -0.716 | | 0.263 | |  |
|  |  | 5-14 Years | | -0.351 | | | | | 0.303 | | -1.16 | | | | 0.247 | | -0.945 | | 0.243 | |  |
|  |  | 15-48 Years | | -0.367 | | | | | 0.322 | | -1.14 | | | | 0.254 | | -0.998 | | 0.264 | |  |
|  |  | 49-59 Years | | -0.355 | | | | | 0.321 | | -1.1 | | | | 0.269 | | -0.985 | | 0.275 | |  |
|  |  | 60 Years and above | | -0.293 | | | | | 0.307 | | -0.95 | | | | 0.34 | | -0.894 | | 0.308 | |  |
| Sex | | Male | | 0 | | | | |  | |  | | | |  | |  | |  | |  |
|  |  | Female | | 0.021 | | | | | 0.066 | | 0.32 | | | | 0.747 | | -0.107 | | 0.15 | |  |
| Education | | Not Literate | | 0 | | | | |  | |  | | | |  | |  | |  | |  |
|  |  | Primary | | 0.023 | | | | | 0.07 | | 0.33 | | | | 0.739 | | -0.114 | | 0.161 | |  |
|  |  | Higher Secondary | | 0.085 | | | | | 0.11 | | 0.77 | | | | 0.444 | | -0.132 | | 0.301 | |  |
|  |  | Graduate or Above | | 0.496 | | | | | 0.206 | | 2.41 | | | | 0.016 | | 0.092 | | 0.9 | |  |
| Social group | | ST | | 0 | | | | |  | |  | | | |  | |  | |  | |  |
|  |  | SC | | 0.239 | | | | | 0.162 | | 1.48 | | | | 0.139 | | -0.078 | | 0.556 | |  |
|  |  | OBC | | 0.274 | | | | | 0.159 | | 1.73 | | | | 0.084 | | -0.036 | | 0.585 | |  |
|  |  | Others | | 0.467 | | | | | 0.182 | | 2.57 | | | | 0.01 | | 0.111 | | 0.823 | |  |
| Year | | 2014 | | 0 | | | | |  | |  | | | |  | |  | |  | |  |
|  |  | 2004 | | 0.881 | | | | | 0.546 | | 1.61 | | | | 0.106 | | -0.188 | | 1.951 | |  |
| Category of disease | | Communicable | | 0 | | | | |  | |  | | | |  | |  | |  | |  |
|  |  | NCD | | 0.552 | | | | | 0.086 | | 6.45 | | | | 0 | | 0.384 | | 0.719 | |  |
|  |  | Maternal | | 0.456 | | | | | 0.112 | | 4.08 | | | | 0 | | 0.237 | | 0.674 | |  |
|  |  | Emergency & Injury | | 0.817 | | | | | 0.105 | | 7.79 | | | | 0 | | 0.611 | | 1.022 | |  |
|  |  | Others | | 0.4 | | | | | 0.092 | | 4.33 | | | | 0 | | 0.219 | | 0.581 | |  |
| Type of hospital | | Public hospital | | 0 | | | | |  | |  | | | |  | |  | |  | |  |
|  |  | Private Hospital | | 1.174 | | | | | 0.08 | | 14.75 | | | | 0 | | 1.018 | | 1.33 | |  |
| Hospital duration | | Less than 3 days | | 0 | | | | |  | |  | | | |  | |  | |  | |  |
|  |  | more than 3 days | | 1.073 | | | | | 0.077 | | 14.03 | | | | 0 | | 0.924 | | 1.223 | |  |
|  | | _cons | | -3.363 | | | | | 0.473 | | -7.11 | | | | 0 | | -4.291 | | -2.436 | |  |
|  | | Wald test of exogeneity: chi2(1) = 3.70 Prob> chi2 = 0.0545 | | | | | | | | | | | | | | | | |  | |  |
| . weakiv | | |  | |  | |  | | | | | |  | | | | | | | |  |
| Test | Statistic | | p-value | | Conf. level | | Conf.Set | | | | | |  | | | | | | | |  |
| AR | chi2(1) =2.81 | | 0.094 | | 95% | | [-.232273,3.39606] | | | | | |  | | | | | | | |  |
| Wald | chi2(1) =2.55 | | 0.11 | | 95% | | [-.319493,3.1344] | | | | | |  | | | | | | | |  |
|  |  |  |  | |  |  | |  | |  | |  | |  | |  | |  | |  | |
|  |  |  |  | |  |  | |  | |  | |  | |  | |  | |  | |  | |
| **Table S5.2: IV PROBIT regression to find predictors of CHE25 – Karnataka** | | | | | | | | | | | | | | | | | | |  | |  |
| Two-step Probit with endogenous regressors (IV: Social Group) Number of obs= 4,107 | | | | | | | | | | | | | | | | | | |  | |  |
| Variable | Category | | Coef. | Std. Err. | | | | | z | | P>z | | [95% Conf.Interval] | | | | | |  | |  |
| Government insurance (Instrumented) | Yes | | 2.463 | 2.279 | | | | | 1.08 | | 0.28 | | -2.003 | | | | 6.929 | |  | |  |
| Education | Not Literate | | 0 |  | | | | |  | |  | |  | | | |  | |  | |  |
|  | Primary | | 0.132 | 0.079 | | | | | 1.67 | | 0.1 | | -0.023 | | | | 0.287 | |  | |  |
|  | Higher Secondary | | 0.112 | 0.096 | | | | | 1.16 | | 0.25 | | -0.077 | | | | 0.3 | |  | |  |
|  | Graduate or Above | | 0.257 | 0.128 | | | | | 2.01 | | 0.04 | | 0.007 | | | | 0.508 | |  | |  |
| Quintile | Poorest | | 0 |  | | | | |  | |  | |  | | | |  | |  | |  |
|  | Poor | | -0.38 | 0.1 | | | | | -3.79 | | 0 | | -0.577 | | | | -0.184 | |  | |  |
|  | Middle | | -0.648 | 0.103 | | | | | -6.32 | | 0 | | -0.849 | | | | -0.447 | |  | |  |
|  | Rich | | -1.006 | 0.125 | | | | | -8.05 | | 0 | | -1.25 | | | | -0.761 | |  | |  |
|  | Richest | | -1.418 | 0.151 | | | | | -9.37 | | 0 | | -1.715 | | | | -1.122 | |  | |  |
| Age | <1 year | | 0 |  | | | | |  | |  | |  | | | |  | |  | |  |
|  | 1-4 Years | | -0.084 | 0.289 | | | | | -0.29 | | 0.77 | | -0.651 | | | | 0.483 | |  | |  |
|  | 5-14 Years | | -0.163 | 0.284 | | | | | -0.57 | | 0.57 | | -0.72 | | | | 0.394 | |  | |  |
|  | 15-48 Years | | -0.032 | 0.264 | | | | | -0.12 | | 0.9 | | -0.55 | | | | 0.486 | |  | |  |
|  | 49-59 Years | | 0.133 | 0.287 | | | | | 0.46 | | 0.64 | | -0.43 | | | | 0.697 | |  | |  |
|  | 60 Years and above | | 0.047 | 0.269 | | | | | 0.18 | | 0.86 | | -0.481 | | | | 0.575 | |  | |  |
| Sex | Male | | 0 |  | | | | |  | |  | |  | | | |  | |  | |  |
|  | Female | | -0.075 | 0.065 | | | | | -1.15 | | 0.25 | | -0.201 | | | | 0.052 | |  | |  |
| Place | Rural | | 0 |  | | | | |  | |  | |  | | | |  | |  | |  |
|  | Urban | | -0.022 | 0.066 | | | | | -0.33 | | 0.74 | | -0.151 | | | | 0.108 | |  | |  |
| Year | 2014 | |  |  | | | | |  | |  | |  | | | |  | |  | |  |
|  | 2004 | | 0.008 | 0.167 | | | | | 0.05 | | 0.96 | | -0.319 | | | | 0.334 | |  | |  |
| Category of disease | Communicable | | 0 |  | | | | |  | |  | |  | | | |  | |  | |  |
|  | NCD | | 0.829 | 0.095 | | | | | 8.72 | | 0 | | 0.643 | | | | 1.015 | |  | |  |
|  | Maternal | | 0.779 | 0.109 | | | | | 7.16 | | 0 | | 0.566 | | | | 0.992 | |  | |  |
|  | Emergency & Injury | | 1.12 | 0.123 | | | | | 9.13 | | 0 | | 0.879 | | | | 1.36 | |  | |  |
|  | Others | | 0.613 | 0.101 | | | | | 6.05 | | 0 | | 0.414 | | | | 0.812 | |  | |  |
| Type of hospital | Public hospital | | 0 |  | | | | |  | |  | |  | | | |  | |  | |  |
|  | Private Hospital | | 1.382 | 0.1 | | | | | 13.82 | | 0 | | 1.186 | | | | 1.578 | |  | |  |
| Hospital duration | Less than 3 days | | 0 |  | | | | |  | |  | |  | | | |  | |  | |  |
|  | more than 3 days | | 1.162 | 0.079 | | | | | 14.7 | | 0 | | 1.007 | | | | 1.317 | |  | |  |
|  | _cons | | -2.967 | 0.288 | | | | | -10.31 | | 0 | | -3.532 | | | | -2.403 | |  | |  |
|  | Wald test of exogeneity: chi2(1) = 1.38 Prob> chi2 = 0.2398 | | | | | | | | | | | | | | | |  | |  | |  |
| . weakiv | |  |  | |  | |  | | | | | | | |  | | | |  | |  |
| Test | | Statistic | p-value | | Conf. level | | Conf.Set | | | | | | | |  | | | |  | |  |
| CLR | | stat(.) =1.51 | 0.252 | | 95% | | [-2.13818,11.2149] | | | | | | | |  | | | |  | |  |
| K | | chi2(1) =1.28 | 0.257 | | 95% | | [-2.31863,... ] | | | | | | | |  | | | |  | |  |
| J | | chi2(2) =2.5 | 0.287 | | 95% | | entire grid | | | | | | | |  | | | |  | |  |
| K-J | |  | 0.305 | | 95% (96%,99%) | | [-2.49908,... ] | | | | | | | |  | | | |  | |  |
| AR | | chi2(3) =3.78 | 0.286 | | 95% | | [-2.85997,... ] | | | | | | | |  | | | |  | |  |
| Wald | | chi2(1) =1.17 | 0.28 | | 95% | | [-2.00285,6.92929] | | | | | | | |  | | | |  | |  |
|  |  |  |  | |  |  | |  | |  | |  | |  | |  | |  | |  | |
|  |  |  |  | |  |  | |  | |  | |  | |  | |  | |  | |  | |
| **Table S5.3: IV PROBIT regression to find predictors of CHE25 – Tamil Nadu** | | | | | | | | | | | | | | | | | | | | |  |
| Two-step Probit with endogenous regressors (IV: Place) Number of obs= 5,933 | | | | | | | | | | | | | | | | | | | | |  |
| Variable | | Category | | | Coef. | | | | Std. Err. | | z | | P>z | | | | [95% Conf. | | Interval] | |  |
| Government insurance (Instrumented) | | Yes | | | 1.58 | | | | 1.859 | | 0.85 | | 0.395 | | | | -2.063 | | 5.223 | |  |
| Education | | Not Literate | | | 0 | | | |  | |  | |  | | | |  | |  | |  |
|  |  | Primary | | | 0.063 | | | | 0.068 | | 0.93 | | 0.351 | | | | -0.07 | | 0.196 | |  |
|  |  | Higher Secondary | | | 0.145 | | | | 0.086 | | 1.68 | | 0.093 | | | | -0.024 | | 0.313 | |  |
|  |  | Graduate or Above | | | 0.349 | | | | 0.1 | | 3.49 | | 0 | | | | 0.153 | | 0.546 | |  |
| Quintile | | Poorest | | | 0 | | | |  | |  | |  | | | |  | |  | |  |
|  |  | Poor | | | -0.135 | | | | 0.094 | | -1.44 | | 0.15 | | | | -0.32 | | 0.049 | |  |
|  |  | Middle | | | -0.309 | | | | 0.092 | | -3.37 | | 0.001 | | | | -0.488 | | -0.129 | |  |
|  |  | Rich | | | -0.632 | | | | 0.089 | | -7.1 | | 0 | | | | -0.807 | | -0.458 | |  |
|  |  | Richest | | | -0.983 | | | | 0.108 | | -9.11 | | 0 | | | | -1.194 | | -0.771 | |  |
| Social group | | ST | | | 0 | | | |  | |  | |  | | | |  | |  | |  |
|  |  | SC | | | -0.576 | | | | 0.244 | | -2.36 | | 0.018 | | | | -1.055 | | -0.097 | |  |
|  |  | OBC | | | -0.416 | | | | 0.235 | | -1.77 | | 0.076 | | | | -0.876 | | 0.044 | |  |
|  |  | Others | | | -0.517 | | | | 0.298 | | -1.73 | | 0.083 | | | | -1.101 | | 0.068 | |  |
| Age | | <1 year | | | 0 | | | |  | |  | |  | | | |  | |  | |  |
|  |  | 1-4 Years | | | -0.311 | | | | 0.261 | | -1.19 | | 0.234 | | | | -0.822 | | 0.2 | |  |
|  |  | 5-14 Years | | | -0.186 | | | | 0.242 | | -0.77 | | 0.442 | | | | -0.659 | | 0.288 | |  |
|  |  | 15-48 Years | | | 0.037 | | | | 0.22 | | 0.17 | | 0.866 | | | | -0.393 | | 0.467 | |  |
|  |  | 49-59 Years | | | 0.04 | | | | 0.247 | | 0.16 | | 0.871 | | | | -0.444 | | 0.524 | |  |
|  |  | 60 Years and above | | | 0.139 | | | | 0.217 | | 0.64 | | 0.52 | | | | -0.286 | | 0.564 | |  |
| Sex | | Male | | | 0 | | | |  | |  | |  | | | |  | |  | |  |
|  |  | Female | | | -0.084 | | | | 0.054 | | -1.55 | | 0.12 | | | | -0.189 | | 0.022 | |  |
| Year | | 2014 | | | 0 | | | |  | |  | |  | | | |  | |  | |  |
|  |  | 2004 | | | 0.223 | | | | 0.358 | | 0.62 | | 0.534 | | | | -0.48 | | 0.925 | |  |
| Category of disease | | Communicable | | | 0 | | | |  | |  | |  | | | |  | |  | |  |
|  |  | NCD | | | 0.704 | | | | 0.087 | | 8.05 | | 0 | | | | 0.533 | | 0.875 | |  |
|  |  | Maternal | | | 0.646 | | | | 0.115 | | 5.63 | | 0 | | | | 0.421 | | 0.871 | |  |
|  |  | Emergency & Injury | | | 0.75 | | | | 0.102 | | 7.38 | | 0 | | | | 0.551 | | 0.949 | |  |
|  |  | Others | | | 0.539 | | | | 0.086 | | 6.25 | | 0 | | | | 0.37 | | 0.708 | |  |
| Type of hospital | | Public hospital | | | 0 | | | |  | |  | |  | | | |  | |  | |  |
|  |  | Private Hospital | | | 2.101 | | | | 0.097 | | 21.7 | | 0 | | | | 1.91 | | 2.291 | |  |
| Hospital duration | | Less than 3 days | | | 0 | | | |  | |  | |  | | | |  | |  | |  |
|  |  | more than 3 days | | | 1.161 | | | | 0.067 | | 17.2 | | 0 | | | | 1.029 | | 1.293 | |  |
|  | | _cons | | | -3.405 | | | | 0.43 | | -7.92 | | 0 | | | | -4.248 | | -2.562 | |  |
|  | | Wald test of exogeneity: chi2(1) = 0.80 Prob> chi2 = 0.3702 | | | | | | | | | | | | | | | | |  | |  |
| . weakiv |  | |  |  | | |  | | | | | | | |  | | | | | |  |
| Test | Statistic | | p-value | Conf. level | | | Conf.Set | | | | | | | |  | | | | | |  |
| AR | chi2(1) =0.77 | | 0.379 | 95% | | | [-2.02639,6.95294] | | | | | | | |  | | | | | |  |
| Wald | chi2(1) =0.72 | | 0.395 | 95% | | | [-2.06319,5.22331] | | | | | | | |  | | | | | |  |
