# Supplementary material for: Utilisation and financial protection for hospital care under publicly funded health insurance in three states in Southern India
Source: BMC Health Serv Res. 2019 Dec 27;19:1004. doi: 10.1186/s12913-019-4849-8 (PMC6935172; doi:10.1186/s12913-019-4849-8)
Supplement: Supplementary file 6 — Additional file 6. IVPROBIT Regression for CHE40. [file 12913_2019_4849_MOESM6_ESM.docx]

| **Additional file 6- IVPROBIT Regression for CHE40** | | | | |  |  |  |
| --- | --- | --- | --- | --- | --- | --- | --- |
|  |  |  |  |  |  |  |  |
| **Table S6.1: IV PROBIT regression to find out predictors of CHE40 - Andhra Pradesh** | | | | | |  |  |
|  |  |  |  |  |  |  |  |
|  | Two-step probit with endogenous regressors (IV - Social Group) Number of obs = 4,520 | | | | | | |
| Variable | Category | Coef. | Std. Err. | z | P>z | [95% Conf.Interval] | |
| Government insurance | Yes | -1.788 | 1.171 | -1.530 | 0.127 | -4.084 | 0.508 |
| Education | Not Literate | 0 |  |  |  |  |  |
|  | Primary | 0.060 | 0.083 | 0.730 | 0.467 | -0.102 | 0.222 |
|  | Higher Secondary | 0.085 | 0.134 | 0.630 | 0.528 | -0.178 | 0.348 |
|  | Graduate or Above | -0.072 | 0.257 | -0.280 | 0.778 | -0.576 | 0.431 |
| Quintile | Poorest | 0 |  |  |  |  |  |
|  | Poor | -0.349 | 0.101 | -3.460 | 0.001 | -0.546 | -0.151 |
|  | Middle | -0.476 | 0.132 | -3.620 | 0.000 | -0.734 | -0.218 |
|  | Rich | -0.723 | 0.144 | -5.030 | 0.000 | -1.005 | -0.441 |
|  | Richest | -0.843 | 0.127 | -6.620 | 0.000 | -1.092 | -0.593 |
| Age | <1 year | 0 |  |  |  |  |  |
|  | 1-4 Years | 0.316 | 0.311 | 1.020 | 0.310 | -0.294 | 0.926 |
|  | 5-14 Years | 0.316 | 0.389 | 0.810 | 0.417 | -0.446 | 1.077 |
|  | 15-48 Years | 0.553 | 0.411 | 1.340 | 0.179 | -0.253 | 1.359 |
|  | 49-59 Years | 0.549 | 0.411 | 1.330 | 0.182 | -0.257 | 1.355 |
|  | 60 Years and above | 0.510 | 0.385 | 1.320 | 0.185 | -0.245 | 1.265 |
| Sex | Male | 0 |  |  |  |  |  |
|  | Female | -0.094 | 0.077 | -1.220 | 0.221 | -0.244 | 0.056 |
| Place | Rural | 0 |  |  |  |  |  |
|  | Urban | -0.221 | 0.112 | -1.980 | 0.047 | -0.440 | -0.003 |
| Year | 2014 |  |  |  |  |  |  |
|  | 2004 | -1.001 | 0.728 | -1.380 | 0.169 | -2.428 | 0.425 |
| Category of disease | Communicable | 0 |  |  |  |  |  |
|  | NCD | 0.520 | 0.101 | 5.130 | 0.000 | 0.321 | 0.718 |
|  | Maternal | 0.013 | 0.137 | 0.090 | 0.927 | -0.257 | 0.282 |
|  | Emergency & Injury | 0.733 | 0.120 | 6.090 | 0.000 | 0.497 | 0.969 |
|  | Others | 0.330 | 0.110 | 2.990 | 0.003 | 0.113 | 0.546 |
| Type of hospital | Public hospital | 0 |  |  |  |  |  |
|  | Private Hospital | 0.973 | 0.107 | 9.100 | 0.000 | 0.763 | 1.182 |
| Hospital duration | Less than 3 days | 0 |  |  |  |  |  |
|  | more than 3 days | 1.065 | 0.097 | 11.020 | 0.000 | 0.876 | 1.254 |
|  | _cons | -2.200 | 0.521 | -4.230 | 0.000 | -3.220 | -1.179 |
|  |  |  |  |  |  |  |  |
|  | Wald test of exogeneity: chi2(1) = 1.80 Prob > chi2 = 0.1800 | | | | |  |  |
|  |  |  |  |  |  |  |  |
|  | . weakiv |  |  |  |  |  |  |
|  |  |  |  |  |  |  |  |
|  | Test | Statistic | p-value | Conf. level | Conf.Set |  |  |
|  | CLR | stat(.) =2.57 | 0.122 | 95% | [-4.70967,.484872] |  |  |
|  | K | chi2(1) =2.46 | 0.117 | 95% | [-4.70967,.484872] |  |  |
|  | J | chi2(2) =1.37 | 0.503 | 95% | entire grid |  |  |
|  | K-J |  | 0.143 | 95% (96%,99%) | [-4.89519,.577631] |  |  |
|  | AR | chi2(3) =3.83 | 0.281 | 95% | [-5.91555,1.13419] |  |  |
|  | Wald | chi2(1) =2.33 | 0.127 | 95% | [-4.08354,.508062] |  |  |
|  |  |  |  |  |  |  |  |
|  |  |  |  |  |  |  |  |
| **Table S6.2: IV PROBIT regression to find out predictors of CHE40 - Karnataka** | | | | |  |  |  |
|  |  |  |  |  |  |  |  |
|  | Two-step probit with endogenous regressors (IV - Social Group) Number of obs = 4,107 | | | | | | |
| Variable | Category | Coef. | Std. Err. | z | P>z | [95% Conf.Interval] | |
| Govt insurance | Yes | 0.788 | 2.668 | 0.300 | 0.768 | -4.440 | 6.016 |
| Education | Not Literate | 0 |  |  |  |  |  |
|  | Primary | 0.141 | 0.090 | 1.570 | 0.116 | -0.035 | 0.317 |
|  | Higher Secondary | 0.163 | 0.110 | 1.490 | 0.137 | -0.052 | 0.378 |
|  | Graduate or Above | 0.305 | 0.144 | 2.120 | 0.034 | 0.023 | 0.587 |
| Quintile | Poorest | 0 |  |  |  |  |  |
|  | Poor | -0.461 | 0.107 | -4.330 | 0.000 | -0.670 | -0.252 |
|  | Middle | -0.841 | 0.112 | -7.480 | 0.000 | -1.061 | -0.621 |
|  | Rich | -1.134 | 0.142 | -7.980 | 0.000 | -1.413 | -0.856 |
|  | Richest | -1.387 | 0.172 | -8.040 | 0.000 | -1.725 | -1.049 |
| Age | <1 year | 0 |  |  |  |  |  |
|  | 1-4 Years | -0.583 | 0.346 | -1.680 | 0.093 | -1.262 | 0.096 |
|  | 5-14 Years | -0.362 | 0.316 | -1.150 | 0.252 | -0.981 | 0.257 |
|  | 15-48 Years | -0.186 | 0.289 | -0.640 | 0.519 | -0.753 | 0.380 |
|  | 49-59 Years | -0.062 | 0.318 | -0.190 | 0.846 | -0.685 | 0.561 |
|  | 60 Years and above | -0.025 | 0.295 | -0.090 | 0.931 | -0.603 | 0.552 |
| Sex | Male | 0 |  |  |  |  |  |
|  | Female | -0.086 | 0.073 | -1.180 | 0.239 | -0.230 | 0.057 |
| Place | Rural | 0 |  |  |  |  |  |
|  | Urban | 0.069 | 0.076 | 0.910 | 0.364 | -0.080 | 0.218 |
| Year | 2014 | 0 |  |  |  |  |  |
|  | 2004 | -0.138 | 0.193 | -0.710 | 0.476 | -0.515 | 0.240 |
| Category of disease | Communicable | 0 |  |  |  |  |  |
|  | NCD | 0.750 | 0.113 | 6.640 | 0.000 | 0.529 | 0.971 |
|  | Maternal | 0.489 | 0.131 | 3.740 | 0.000 | 0.233 | 0.745 |
|  | Emergency & Injury | 1.044 | 0.138 | 7.550 | 0.000 | 0.773 | 1.315 |
|  | Others | 0.490 | 0.122 | 4.010 | 0.000 | 0.250 | 0.729 |
| Type of hospital | Public hospital | 0 |  |  |  |  |  |
|  | Private Hospital | 1.184 | 0.122 | 9.700 | 0.000 | 0.944 | 1.423 |
| Hospital duration | Less than 3 days | 0 |  |  |  |  |  |
|  | more than 3 days | 1.169 | 0.106 | 11.030 | 0.000 | 0.961 | 1.376 |
|  | _cons | -2.877 | 0.321 | -8.970 | 0.000 | -3.506 | -2.248 |
|  |  |  |  |  |  |  |  |
|  | Wald test of exogeneity: chi2(1) = 0.10 Prob > chi2 = 0.7578 | | | | |  |  |
|  |  |  |  |  |  |  |  |
|  | . weakiv |  |  |  |  |  |  |
|  |  |  |  |  |  |  |  |
|  | Test | Statistic | p-value | Conf. level | Conf.Set |  |  |
|  | CLR | stat(.) =0.15 | 0.718 | 95% | [-8.40126,10.8218] |  |  |
|  | K | chi2(1) =0.1 | 0.754 | 95% | entire grid |  |  |
|  | J | chi2(2) =5.39 | 0.068 | 95% | [-2.90895,6.38573] |  |  |
|  | K-J |  | 0.320 | 95% (96%,99%) | entire grid |  |  |
|  | AR | chi2(3) =5.49 | 0.139 | 95% | [-4.38765, 7.44194] |  |  |
|  | Wald | chi2(1) =0.09 | 0.768 | 95% | [-4.44046,6.01605] |  |  |
|  |  |  |  |  |  |  |  |
|  |  |  |  |  |  |  |  |
| **Table S6.3: IV PROBIT regression to find out predictors of CHE40 - Tamil Nadu** | | | | | |  |  |
|  |  |  |  |  |  |  |  |
|  | Two-step probit with endogenous regressors (IV - Place) Number of obs = 5,933 | | | | | | |
| Variable | Category | Coef. | Std. Err. | z | P>z | [95% Conf.Interval] | |
| Govt insurance | Yes | 1.653 | 2.099 | 0.790 | 0.431 | -2.462 | 5.768 |
| Education | Not Literate | 0 |  |  |  |  |  |
|  | Primary | 0.079 | 0.075 | 1.050 | 0.294 | -0.069 | 0.227 |
|  | Higher Secondary | 0.083 | 0.098 | 0.840 | 0.399 | -0.109 | 0.275 |
|  | Graduate or Above | 0.385 | 0.112 | 3.430 | 0.001 | 0.165 | 0.604 |
| Quintile | Poorest | 0 |  |  |  |  |  |
|  | Poor | -0.104 | 0.102 | -1.020 | 0.308 | -0.303 | 0.096 |
|  | Middle | -0.300 | 0.100 | -3.010 | 0.003 | -0.495 | -0.105 |
|  | Rich | -0.637 | 0.098 | -6.470 | 0.000 | -0.830 | -0.444 |
|  | Richest | -0.992 | 0.121 | -8.200 | 0.000 | -1.230 | -0.755 |
| Social group | ST | 0 |  |  |  |  |  |
|  | SC | -0.364 | 0.267 | -1.360 | 0.173 | -0.888 | 0.159 |
|  | OBC | -0.308 | 0.256 | -1.200 | 0.229 | -0.810 | 0.193 |
|  | Others | -0.272 | 0.329 | -0.830 | 0.408 | -0.917 | 0.372 |
| Age | <1 year | 0 |  |  |  |  |  |
|  | 1-4 Years | -0.616 | 0.298 | -2.070 | 0.039 | -1.200 | -0.032 |
|  | 5-14 Years | -0.654 | 0.271 | -2.410 | 0.016 | -1.185 | -0.122 |
|  | 15-48 Years | -0.251 | 0.234 | -1.070 | 0.284 | -0.709 | 0.208 |
|  | 49-59 Years | -0.258 | 0.267 | -0.970 | 0.333 | -0.781 | 0.265 |
|  | 60 Years and above | -0.145 | 0.231 | -0.630 | 0.531 | -0.597 | 0.308 |
| Sex | Male | 0 |  |  |  |  |  |
|  | Female | -0.117 | 0.060 | -1.940 | 0.052 | -0.235 | 0.001 |
| Year | 2014 | 0 |  |  |  |  |  |
|  | 2004 | 0.290 | 0.404 | 0.720 | 0.473 | -0.502 | 1.083 |
| Category of disease | Communicable | 0 |  |  |  |  |  |
|  | NCD | 0.719 | 0.102 | 7.060 | 0.000 | 0.520 | 0.919 |
|  | Maternal | 0.480 | 0.135 | 3.570 | 0.000 | 0.216 | 0.745 |
|  | Emergency & Injury | 0.798 | 0.115 | 6.910 | 0.000 | 0.572 | 1.024 |
|  | Others | 0.591 | 0.101 | 5.880 | 0.000 | 0.394 | 0.788 |
| Type of hospital | Public hospital | 0 |  |  |  |  |  |
|  | Private Hospital | 1.735 | 0.113 | 15.300 | 0.000 | 1.513 | 1.958 |
| Hospital duration | Less than 3 days | 0 |  |  |  |  |  |
|  | more than 3 days | 1.005 | 0.081 | 12.370 | 0.000 | 0.846 | 1.164 |
|  | _cons | -3.310 | 0.477 | -6.940 | 0.000 | -4.244 | -2.375 |
|  |  |  |  |  |  |  |  |
|  | Wald test of exogeneity: chi2(1) = 0.58 Prob > chi2 = 0.4459 | | | | |  |  |
|  |  |  |  |  |  |  |  |
|  | . weakiv |  |  |  |  |  |  |
|  |  |  |  |  |  |  |  |
|  | Test | Statistic | p-value | Conf. level | Conf.Set |  |  |
|  | AR | chi2(1) =0.65 | 0.419 | 95% | [-2.5864,7.55548] |  |  |
|  | Wald | chi2(1) =0.62 | 0.431 | 95% | [-2.4617,5.76818] |  |  |
