# Supplementary material for: Utilisation and financial protection for hospital care under publicly funded health insurance in three states in Southern India
Source: BMC Health Serv Res. 2019 Dec 27;19:1004. doi: 10.1186/s12913-019-4849-8 (PMC6935172; doi:10.1186/s12913-019-4849-8)
Supplement: Supplementary file 7 — Additional file 7. Incidence of CHE10. [file 12913_2019_4849_MOESM7_ESM.docx]

**Additional file 7– Incidence of CHE10**

**Table S7: Proportion of individuals incurred CHE10 for Hospitalisation Episode (%) with 95% confidence intervals in ( )**

| State | For PFHI Enrolled in 2014 | | For Non-enrolled in 2014 | | 2004 | |
| --- | --- | --- | --- | --- | --- | --- |
|  | Public | Private | Public | Private | Public | Private |
| Andhra Pradesh | 8.7  (5.8-11.6) | 51  (47.8-54.2) | 7.3  (3.5-11.2) | 50.9  (47.4-54.4) | 17.9  (15.1-20.7) | 53.6  (51.2 – 56.1) |
| Karnataka | 8  (1.4-14.5) | 43.1  (34.5-51.7) | 11.5  (9.3-13.9) | 53.2  (50.9-55.5) | 20.3  (16.8-23.8) | 49.6  (46.5-52.8) |
| Tamil Nadu | 0.7  (0-1.9) | 59.3  (54.7-63.9) | 1.2  (0.6-1.8) | 58.3  (55.9-60.6) | 8  (6.3-9.7) | 50  (47.4-52.5) |
