# Supplementary material for: Utilisation and financial protection for hospital care under publicly funded health insurance in three states in Southern India
Source: BMC Health Serv Res. 2019 Dec 27;19:1004. doi: 10.1186/s12913-019-4849-8 (PMC6935172; doi:10.1186/s12913-019-4849-8)
Supplement: Supplementary file 8 — Additional file 8. IV PROBIT Regression for CHE10. [file 12913_2019_4849_MOESM8_ESM.docx]

| **Additional file 8 - IV PROBIT Regression for CHE10** | | | | | |  |  |
| --- | --- | --- | --- | --- | --- | --- | --- |
|  |  |  |  |  |  |  |  |
| **Table S8.1: IV PROBIT regression to find predictors of CHE10 – Andhra Pradesh** | | | | | |  |  |
| Two-step Probit with endogenous regressors (IV: Social Group) Number of obs= 4,520 | | | | | |  |  |
|  | | | | | | | |
| Variable | Category | Coef. | Std. Err. | Z | P>z | [95% Conf.Interval] | |
| Government insurance (Instrumented) | Yes | -1.35178 | 0.8440585 | -1.6 | 0.109 | -3.006104 | 0.3025442 |
| Education | Not Literate |  |  |  |  |  |  |
|  | Primary | -0.066936 | 0.0616356 | -1.09 | 0.277 | -0.1877395 | 0.0538674 |
|  | Higher Secondary | 0.0168172 | 0.0952217 | 0.18 | 0.86 | -0.1698138 | 0.2034483 |
|  | Graduate or Above | -0.0230561 | 0.1838629 | -0.13 | 0.9 | -0.3834208 | 0.3373087 |
| Quintile | Poorest |  |  |  |  |  |  |
|  | Poor | -0.1994487 | 0.082386 | -2.42 | 0.015 | -0.3609222 | -0.0379752 |
|  | Middle | -0.2518988 | 0.1012655 | -2.49 | 0.013 | -0.4503755 | -0.0534222 |
|  | Rich | -0.5181753 | 0.1068677 | -4.85 | 0 | -0.7276321 | -0.3087185 |
|  | Richest | -0.8603873 | 0.0958539 | -8.98 | 0 | -1.048257 | -0.6725172 |
| Place | Rural |  |  |  |  |  |  |
|  | Urban | -0.244088 | 0.0803711 | -3.04 | 0.002 | -0.4016125 | -0.0865635 |
| Age | <1 year |  |  |  |  |  |  |
|  | 1-4 Years | -0.0822524 | 0.2065771 | -0.4 | 0.691 | -0.487136 | 0.3226312 |
|  | 5-14 Years | 0.2014257 | 0.265823 | 0.76 | 0.449 | -0.3195778 | 0.7224292 |
|  | 15-48 Years | 0.5810434 | 0.2845474 | 2.04 | 0.041 | 0.0233408 | 1.138746 |
|  | 49-59 Years | 0.5118975 | 0.2851454 | 1.8 | 0.073 | -0.0469772 | 1.070772 |
|  | 60 Years and above | 0.4165982 | 0.2653292 | 1.57 | 0.116 | -0.1034374 | 0.9366338 |
| Sex | Male |  |  |  |  |  |  |
|  | Female | -0.0070053 | 0.0557063 | -0.13 | 0.9 | -0.1161877 | 0.102177 |
| Year | 2014 |  |  |  |  |  |  |
|  | 2004 | -0.8970751 | 0.5255331 | -1.71 | 0.088 | -1.927101 | 0.1329509 |
| Category of disease | Communicable |  |  |  |  |  |  |
|  | NCD | 0.3772365 | 0.0665274 | 5.67 | 0 | 0.2468452 | 0.5076279 |
|  | Maternal | 0.2934257 | 0.0917386 | 3.2 | 0.001 | 0.1136213 | 0.4732301 |
|  | Emergency & Injury | 0.5469593 | 0.0882813 | 6.2 | 0 | 0.3739311 | 0.7199874 |
|  | Others | 0.313951 | 0.0740202 | 4.24 | 0 | 0.1688741 | 0.4590279 |
| Type of hospital | Public Hospital |  |  |  |  |  |  |
|  | Private Hospital | 1.410564 | 0.0679077 | 20.77 | 0 | 1.277468 | 1.543661 |
| Hospital duration | Less than 4 days |  |  |  |  |  |  |
|  | More than 4 days | 1.085027 | 0.0553094 | 19.62 | 0 | 0.976623 | 1.193432 |
|  | _cons | -1.39961 | 0.3620767 | -3.87 | 0 | -2.109267 | -0.6899523 |
|  | Wald test of exogeneity: chi2(1) = 1.86 Prob > chi2 = 0.1724 | | | | |  |  |
|  |  |  |  |  |  |  |  |
| .weakiv |  |  |  |  |  |  |  |
| Test | Statistic | p-value | Conf. level | Conf.Set |  |  |  |
| CLR | stat(.) =2.93 | 0.0992 | 95% | [-3.72465,.285834] |  |  |  |
| K | chi2(1) =2.63 | 0.105 | 95% | [-3.79149,.285834] |  |  |  |
| J | chi2(2) =3.06 | 0.2161 | 95% | entiregrid |  |  |  |
| K-J |  | 0.1285 | 95% (96%,99%) | [-3.92517,.352675] |  |  |  |
| AR | chi2(3) =5.69 | 0.1276 | 95% | [-4.05886,.486358] |  |  |  |
| Wald | chi2(1) =2.56 | 0.1093 | 95% | [ -3.0061,.302544] |  |  |  |
|  |  |  |  |  |  |  |  |
|  |  |  |  |  |  |  |  |
|  |  |  |  |  |  |  |  |
|  |  |  |  |  |  |  |  |
|  |  |  |  |  |  |  |  |
|  |  |  |  |  |  |  |  |
|  |  |  |  |  |  |  |  |
| **Table S8.2: IV PROBIT regression to find predictors of CHE10 – Karnatka** | | | | |  |  |  |
|  |  |  |  |  |  |  |  |
| Two-step probit with endogenous regressors (IV: Place) Number of obs = 4,107 | | | | | |  |  |
|  |  |  |  |  |  |  |  |
| Variable | Category | Coef. | Std. Err. | Z | P>z | [95% Conf.Interval] | |
| Government insurance (Instrumented) | Yes | 3.546654 | 6.232684 | 0.57 | 0.569 | -8.669182 | 15.76249 |
| Education | Not Literate |  |  |  |  |  |  |
|  | Primary | 0.0674847 | 0.0716919 | 0.94 | 0.347 | -0.0730289 | 0.2079983 |
|  | Higher Secondary | -0.0283593 | 0.1586048 | -0.18 | 0.858 | -0.339219 | 0.2825004 |
|  | Graduate or Above | 0.1053426 | 0.1840246 | 0.57 | 0.567 | -0.2553389 | 0.4660241 |
| Quintile | Poorest |  |  |  |  |  |  |
|  | Poor | -0.2544628 | 0.1000143 | -2.54 | 0.011 | -0.4504874 | -0.0584383 |
|  | Middle | -0.6713445 | 0.0989921 | -6.78 | 0 | -0.8653654 | -0.4773237 |
|  | Rich | -1.120385 | 0.2321077 | -4.83 | 0 | -1.575308 | -0.6654622 |
|  | Richest | -1.537955 | 0.3328127 | -4.62 | 0 | -2.190256 | -0.8856541 |
| Social group | ST |  |  |  |  |  |  |
|  | SC | 0.0580663 | 0.2416908 | 0.24 | 0.81 | -0.415639 | 0.5317716 |
|  | OBC | 0.0879196 | 0.1533912 | 0.57 | 0.567 | -0.2127217 | 0.3885609 |
|  | Others | 0.0468596 | 0.1220064 | 0.38 | 0.701 | -0.1922686 | 0.2859879 |
| Age | <1 year |  |  |  |  |  |  |
|  | 1-4 Years | -0.267882 | 0.2556317 | -1.05 | 0.295 | -0.7689108 | 0.2331468 |
|  | 5-14 Years | -0.162221 | 0.2692834 | -0.6 | 0.547 | -0.6900067 | 0.3655647 |
|  | 15-48 Years | -0.0779227 | 0.2512305 | -0.31 | 0.756 | -0.5703255 | 0.41448 |
|  | 49-59 Years | -0.1323822 | 0.3858188 | -0.34 | 0.732 | -0.8885732 | 0.6238088 |
|  | 60 Years and above | -0.0829211 | 0.2990148 | -0.28 | 0.782 | -0.6689793 | 0.503137 |
| Sex | Male |  |  |  |  |  |  |
|  | Female | -0.0761948 | 0.0633858 | -1.2 | 0.229 | -0.2004287 | 0.0480392 |
| Year | 2014 |  |  |  |  |  |  |
|  | 2004 | 0.0010185 | 0.392532 | 0 | 0.998 | -0.7683301 | 0.7703672 |
| Category of disease | Communicable |  |  |  |  |  |  |
|  | NCD | 0.8086713 | 0.087207 | 9.27 | 0 | 0.6377488 | 0.9795939 |
|  | Maternal | 0.813996 | 0.1237613 | 6.58 | 0 | 0.5714283 | 1.056564 |
|  | Emergency & Injury | 0.9822996 | 0.1334179 | 7.36 | 0 | 0.7208052 | 1.243794 |
|  | Others | 0.648049 | 0.0868314 | 7.46 | 0 | 0.4778626 | 0.8182355 |
| Type of hospital | Public Hospital |  |  |  |  |  |  |
|  | Private Hospital | 1.567122 | 0.1416561 | 11.06 | 0 | 1.289482 | 1.844763 |
| Hospital duration | Less than 4 days |  |  |  |  |  |  |
|  | More than 4 days | 1.04684 | 0.0738472 | 14.18 | 0 | 0.9021018 | 1.191578 |
|  | _cons | -1.98019 | 0.2735027 | -7.24 | 0 | -2.516245 | -1.444134 |
|  | Wald test of exogeneity: chi2(1) = 0.45 Prob > chi2 = 0.5041 | | | | |  |  |
|  |  |  |  |  |  |  |  |
| weakiv |  |  |  |  |  |  |  |
| Test | Statistic | p-value | Conf. level | Conf.Set |  |  |  |
| AR | chi2(1) =0.41 | 0.5218 | 95% | entire grid |  |  |  |
| Wald | chi2(1) =0.32 | 0.5693 | 95% | [-8.66918,15.7625] |  |  |  |
|  |  |  |  |  |  |  |  |
|  |  |  |  |  |  |  |  |
|  |  |  |  |  |  |  |  |
|  |  |  |  |  |  |  |  |
|  |  |  |  |  |  |  |  |
|  |  |  |  |  |  |  |  |
| **Table S8.3: IV PROBIT regression to find predictors of CHE10 – Tamil Nadu** | | | | |  |  |  |
|  |  |  |  |  |  |  |  |
| Two-step probit with endogenous regressors (IV: Age) Number of obs = 5,933 | | | | | |  |  |
|  |  |  |  |  |  |  |  |
| Variable | Category | Coef. | Std. Err. | Z | P>z | [95% Conf.Interval] | |
| Government insurance (Instrumented) | Yes | 1.039547 | 1.048903 | 0.99 | 0.322 | -1.016266 | 3.09536 |
| Education | Not Literate |  |  |  |  |  |  |
|  | Primary | 0.0053565 | 0.0572267 | 0.09 | 0.925 | -0.1068059 | 0.1175188 |
|  | Higher Secondary | 0.1732749 | 0.071754 | 2.41 | 0.016 | 0.0326397 | 0.3139101 |
|  | Graduate or Above | 0.3624385 | 0.085243 | 4.25 | 0 | 0.1953652 | 0.5295118 |
| Quintile | Poorest |  |  |  |  |  |  |
|  | Poor | 0.0145758 | 0.080862 | 0.18 | 0.857 | -0.1439107 | 0.1730624 |
|  | Middle | -0.2400682 | 0.0805612 | -2.98 | 0.003 | -0.3979652 | -0.0821712 |
|  | Rich | -0.3983885 | 0.0800008 | -4.98 | 0 | -0.5551872 | -0.2415898 |
|  | Richest | -0.7526604 | 0.0934466 | -8.05 | 0 | -0.9358123 | -0.5695084 |
| Social group | ST |  |  |  |  |  |  |
|  | SC | -0.559128 | 0.2163183 | -2.58 | 0.01 | -0.983104 | -0.135152 |
|  | OBC | -0.4002329 | 0.2109452 | -1.9 | 0.058 | -0.8136779 | 0.013212 |
|  | Others | -0.4968806 | 0.2442362 | -2.03 | 0.042 | -0.9755748 | -0.0181864 |
| Place | Rural |  |  |  |  |  |  |
|  | Urban | -0.1453027 | 0.0555916 | -2.61 | 0.009 | -0.2542603 | -0.0363452 |
| Sex | Male |  |  |  |  |  |  |
|  | Female | -0.113703 | 0.0464591 | -2.45 | 0.014 | -0.2047612 | -0.0226449 |
| Year | 2014 |  |  |  |  |  |  |
|  | 2004 | 0.1157264 | 0.2025831 | 0.57 | 0.568 | -0.2813291 | 0.5127819 |
| Category of disease | Communicable |  |  |  |  |  |  |
|  | NCD | 0.5496143 | 0.0689638 | 7.97 | 0 | 0.4144477 | 0.684781 |
|  | Maternal | 0.6587137 | 0.0840828 | 7.83 | 0 | 0.4939145 | 0.8235129 |
|  | Emergency & Injury | 0.6576155 | 0.0864005 | 7.61 | 0 | 0.4882736 | 0.8269574 |
|  | Others | 0.4356784 | 0.0702995 | 6.2 | 0 | 0.297894 | 0.5734629 |
| Type of hospital | Public Hospital |  |  |  |  |  |  |
|  | Private Hospital | 2.359368 | 0.0655638 | 35.99 | 0 | 2.230865 | 2.48787 |
| Hospital duration | Less than 4 days |  |  |  |  |  |  |
|  | More than 4 days | 1.020667 | 0.0499419 | 20.44 | 0 | 0.922783 | 1.118552 |
|  | _cons | -2.509033 | 0.2838437 | -8.84 | 0 | -3.065356 | -1.952709 |
|  | Wald test of exogeneity: chi2(1) = 1.23 Prob > chi2 = 0.2673 | | | | |  |  |
|  |  |  |  |  |  |  |  |
| weakiv |  |  |  |  |  |  |  |
| Test | Statistic | p-value | Conf. level | Conf.Set |  |  |  |
| CLR | stat(.) =1.28 | 0.2964 | 95% | [-1.16163,4.48667] |  |  |  |
| K | chi2(1) =1.05 | 0.3045 | 95% | [-1.24469,4.65279] |  |  |  |
| J | chi2(4) =4.98 | 0.2891 | 95% | entiregrid |  |  |  |
| K-J |  | 0.3575 | 95% (96%,99%) | [-1.32775,4.90198] |  |  |  |
| AR | chi2(5) =6.04 | 0.3027 | 95% | [ -1.66,... ] |  |  |  |
| Wald | chi2(1) =0.98 | 0.3216 | 95% | [-1.01627,3.09536] |  |  |  |
